# Reactivation of a developmentally silenced embryonic globin gene

**DOI:** 10.1038/s41467-021-24402-3

**Published:** 2021-07-21

**Authors:** Andrew J. King, Duantida Songdej, Damien J. Downes, Robert A. Beagrie, Siyu Liu, Megan Buckley, Peng Hua, Maria C. Suciu, A. Marieke Oudelaar, Lars L. P. Hanssen, Danuta Jeziorska, Nigel Roberts, Stephanie J. Carpenter, Helena Francis, Jelena Telenius, Aude-Anais Olijnik, Jacqueline A. Sharpe, Jacqueline Sloane-Stanley, Jennifer Eglinton, Mira T. Kassouf, Stuart H. Orkin, Len A. Pennacchio, James O. J. Davies, Jim R. Hughes, Douglas R. Higgs, Christian Babbs

**Affiliations:** 1 MRC Weatherall Institute of Molecular Medicine, University of Oxford, Oxford, UK; 2 Division of Hematology/Oncology, Department of Pediatrics, Faculty of Medicine, Ramathibodi Hospital, Mahidol University, Bangkok, Thailand; 3 MRC Molecular Haematology Unit, MRC Weatherall Institute of Molecular Medicine, University of Oxford, Oxford, UK; 4 Chinese Academy of Medical Sciences Oxford Institute, University of Oxford, Oxford, UK; 5 Max Planck Institute for Biophysical Chemistry, Göttingen, Germany; 6 MRC WIMM Centre for Computational Biology, MRC Weatherall Institute of Molecular Medicine, Radcliffe Department of Medicine, University of Oxford, Oxford, UK; 7 National Haemoglobinopathy Reference Laboratory, Department of Haematology, Level 4, John Radcliffe Hospital, Oxford, UK; 8 Dana-Farber/Boston Children’s Cancer and Blood Disorders Center, Harvard Medical School and Howard Hughes Medical Institute, Boston, MA USA; 9 Functional Genomics Department, Lawrence Berkeley National Laboratory, Berkeley, CA USA; 10 Comparative Biochemistry Program, University of California, Berkeley, CA USA

**Keywords:** Haematopoietic stem cells, Gene silencing, Gene regulation, Chromatin remodelling, Molecular medicine

## Abstract

The α- and β-globin loci harbor developmentally expressed genes, which are silenced throughout post-natal life. Reactivation of these genes may offer therapeutic approaches for the hemoglobinopathies, the most common single gene disorders. Here, we address mechanisms regulating the embryonically expressed α-like globin, termed ζ-globin. We show that in embryonic erythroid cells, the ζ-gene lies within a ~65 kb sub-TAD (topologically associating domain) of open, acetylated chromatin and interacts with the α-globin super-enhancer. By contrast, in adult erythroid cells, the ζ-gene is packaged within a small (~10 kb) sub-domain of hypoacetylated, facultative heterochromatin within the acetylated sub-TAD and that it no longer interacts with its enhancers. The ζ-gene can be partially re-activated by acetylation and inhibition of histone de-acetylases. In addition to suggesting therapies for severe α-thalassemia, these findings illustrate the general principles by which reactivation of developmental genes may rescue abnormalities arising from mutations in their adult paralogues.

## Abstract (web summary)

Globin loci harbor genes that are expressed embryonically and silenced postnatally. Here the authors show that zeta-globin silencing depends upon selective hypoacetylation of its TAD subdomain, which blocks its interaction with the alpha-globin super-enhancer, and zeta-globin can be reactivated by acetylation.

## Introduction

Patterns of gene expression radically change as multipotent stem cells form specialized somatic cells by a process of lineage commitment, differentiation, and maturation. In addition, even within the same cell system, the underlying transcriptional and epigenetic programs may be quite different during embryonic, fetal, and adult life. Developmental switches in gene expression have been intensively studied during the formation of blood (haematopoiesis), particularly with respect to globin gene expression^1,2^. By understanding the molecular basis of such developmental changes in gene expression, it has recently become feasible to reverse the switch from fetal (γ) to adult (β) globin synthesis^3,4^. This illustrates a general approach to curing some human genetic diseases by switching on normal fetal paralogues while switching off mutated adult genes: an important breakthrough in the application of molecular biology to medicine. Hence, globin gene switching during the formation of red blood cells (erythropoiesis) provides an ideal model for understanding the principles underlying programmed developmental changes in gene expression. Here, we have examined the earliest developmental changes in globin gene expression which occur during the transition between embryonic and fetal life.

In both mice and humans, erythropoiesis occurs in distinct waves^5,6^. First, a cohort of cells originating in the embryonic yolk sac is released into the circulation as the heart begins to beat. This cohort of primitive erythroid cells matures in a semi-synchronous manner and eventually the cells enucleate. As they age, these primitive cells are removed from the circulation. On the basis of work in mice, there appears to be a second, transient wave of hematopoiesis, also originating in the yolk sac, which migrates to the fetal liver and produces both erythroid and myeloid cells^7^. These progenitors are referred to as erythroid myeloid progenitors (EMPs). Erythroid cells derived from EMPs are considered to be early definitive cells. Later in development, cells arising from the splanchnopleura migrate to the floor of the dorsal aorta, where they undergo a transition to definitive hematopoietic stem cells that subsequently contribute to the formation of red blood cells throughout life^8^.

**Fig. 1:**
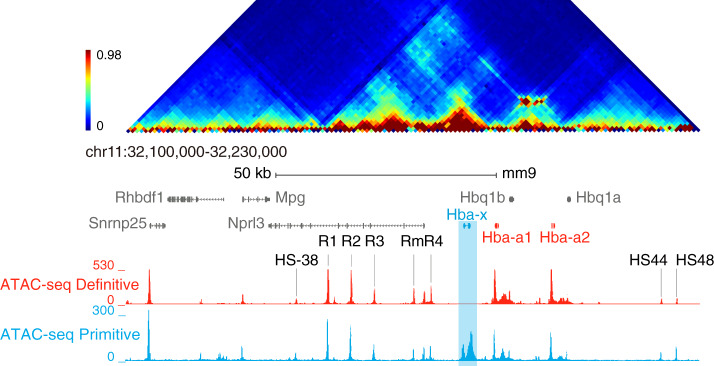
Chromatin environment at the α-globin locus. Upper panel, Tiled-C interaction map showing interactions across the α-globin locus in definitive erythroblasts^16^. Lower, normalized (RPKM) ATAC-seq tracks from primary primitive (blue) and definitive (red) erythroid cells; these were merged from three independent experiments where three litters (primitive) or three fetal livers (definitive) were analyzed in total. The α-globin enhancers (R1–R4;Rm) and the flanking CTCF sites (HS-38, and HS-44;HS-48) of the α-globin gene cluster are indicated. In primitive cells, hypersensitivity is observed over the *Hba-x* gene; the other elements within the gene cluster are unchanged compared to definitive erythroid cells.

Hemoglobin is a heterotetramer comprised of α- and β-like globin chains and, in all mammalian species studied to date, these are encoded by unlinked, multigene loci^9^. Of interest, these multi-gene loci provide examples of co-linearity in which the genes are broadly arranged along the chromosome in the order in which they are expressed during development, in a somewhat similar manner to the developmentally regulated Hox genes^10^. In all cases, globin gene expression is regulated by a cluster of erythroid-specific, distal enhancers lying upstream of the multigene array. In this work, we examine the developmental regulation of the α-globin cluster in mouse and human. In both cases, an embryonic gene (ζ) is linked to duplicated α-genes regulated by 4 (human) or 5 (mouse) distal enhancers (R1–R4; Rm is a mouse specific enhancer) in the order 5′-R1-R2-R3-(Rm)-R4-ζ-α-α−3′ (Fig. 1 and refs. ^11,12^). The ζ-gene is active during embryonic (primitive) erythropoiesis and inactive in all phases of definitive erythropoiesis. The α-globin genes are expressed at all stages of development and their expression relative to ζ-globin increases during primitive erythropoiesis to become the only α-like globin expressed in definitive erythropoiesis^13,14^.

Unlike the fetal globin genes at the β-globin locus, expression of the embryonic ζ-globin gene is almost never re-activated to physiologically relevant levels by *cis*- or *trans*-acting mutations, or during stress erythropoiesis (rapid red-cell expansion occurring, for example, due to hemolytic processes or bleeding), suggesting that this gene is more “deeply” silenced than the fetal (γ) globin genes. Nevertheless, if the ζ-gene could be re-activated, it would complement and compensate for otherwise lethal mutations in the adult α-globin genes^15^.

Here we have examined how the embryonic ζ-globin gene is normally regulated in primitive erythropoiesis and how its silenced state is established and maintained in definitive erythropoiesis. We also show how the ζ-gene may be reactivated in definitive erythropoiesis and address the general question of how genes may be silenced and maintained in a quiescent state, even in the presence of their cognate transcriptional activators and in very close proximity to fully active enhancers.

## Results

### Chromatin accessibility and transcription factor binding

We have previously shown that all sequences required to produce fully regulated expression of the mouse and human ζ- and α-globin genes are contained within a ~65 kb sub-TAD (topologically associating chromatin domain) (Fig. 1 and refs. ^16,17^). This region encompasses a human/mouse syntenic region which contains the entire α-globin cluster linked to *Nprl3*, a widely expressed gene encoding regulator of the mTOR pathway^18^. The *cis*-elements within this region, which are active in definitive erythropoiesis, have been previously identified by ATAC-seq and characterized by ChIP-seq to establish their chromatin signatures^12,19,20^. In this way, we have identified and characterized the promoters, enhancers, and chromatin boundary elements, which together regulate the expression of the α-like globin genes in definitive cells.

Since both the ζ-and α-globin genes are expressed in primitive erythroid cells in a ratio of ~0.4:0.6 respectively, prior to E12.5 when ζ-globin is being silenced (Supplementary Fig. 1)^14^, we initially compared ATAC-seq across the mouse α-globin cluster and its flanking regions in primary primitive and fetal definitive erythroblasts (see the “Methods” section and Supplementary Fig. 2). ATAC-seq showed that all five *cis*-regulatory elements (R1–R4, and Rm) known to be present in definitive mouse erythroid cells^12^ are also found in primitive erythroblasts (Fig. 1); no novel embryonic *cis*-elements were identified. However, in contrast to definitive cells, open chromatin was seen over the ζ-globin gene in primitive cells consistent with its activation and transcription in these cells. A large peak was visible over the ζ-globin promoter and a further peak can be seen extending approximately 1500 bp beyond the 3′ region of the gene.

Next, we performed DNaseI digital footprinting in primitive and definitive erythroid cells. Following DNAse I digestion and deep high-throughput sequencing, regions bound by proteins are protected from DNAseI digestion creating a “footprint” of protected chromatin. The underlying DNA sequence of protected regions can be determined computationally^21^. At the ζ-globin promoter in primitive erythroid cells, evolutionarily conserved consensus binding sequences of the master erythroid factor Gata1, together with binding sequences including CCAT and CACC sites and others that are potentially bound by other more widely expressed factors (Supplementary Table 1) are protected. Using ChIP-seq we confirmed that Gata1 binds the ζ-globin promoter in primitive erythroid cells (Supplementary Fig. 3), the presence of other transcription factors during primitive erythropoiesis is an interesting question that will require further experiments. By contrast, we found no ATAC peak, no DNAseI footprint and no Gata1 binding in definitive cells. Since Gata1 is abundant in definitive cells, it may require a region of accessible chromatin to bind at the globin genes and this is not present at the ζ-globin promoter in definitive cells. DNaseI digital footprints at the known regulatory elements were the same as seen in definitive erythroid cells (Supplementary Fig. 3).

Together these findings show that despite the presence of key erythroid-specific factors in definitive cells, and the close physical proximity of the α-globin enhancer elements, the ζ-globin promoter appears to lie within inaccessible chromatin and does not appear to be bound by Gata1 or any other transcription factors; the latter may relate to the inaccessibility of the DNA wrapped within chromatin.

### Regulation of ζ-globin in primitive erythroid cells

Previous studies in transgenic mice showed that a randomly inserted 4.4 kb segment of DNA containing the entire human ζ-gene with ~559 bp of 5′ flanking and 2184 bp of 3′ flanking DNA is expressed in embryonic erythropoiesis and silenced in definitive erythropoiesis. Furthermore, it was shown that the 5′ flanking region, the body of the ζ-gene and the 3′ non-coding region all contribute to silencing^22^, and that a ~200 bp region of the promoter is necessary for adequate ζ-globin expression^23^. The importance of the normal chromosomal context and the associated distal enhancers was unknown.

Previously, we have shown in definitive erythroid cells that a cluster of five strong, tissue-specific individual enhancer elements in mice (R1–R4 and Rm) fulfill the definition of a super-enhancer, a cluster of strong tissue-specific enhancer elements^12^. In definitive cells, each enhancer acts independently and in an additive fashion, with ~90% of α-globin transcriptional output depending on the R1 and R2 elements alone. Here, we addressed whether the enhancers regulate ζ-globin expression in the same way that they regulate α-globin expression.

To determine whether the five enhancer elements comprise a super-enhancer in primitive erythroid cells, as in definitive cells, we classified accessible regions identified by ATAC-seq into 10 categories using the GenoSTAN Hidden Markov Model^24^ (Supplementary Fig. 4) on the basis of the read-counts for their associated histone marks and CTCF. Individual enhancer regions lying within 12.5 kb of each other were “stitched” together to form a single genomic region and then ranked based upon H3K27ac signal^25^ (Supplementary Fig. 4). As in definitive erythroid cells, the α- and β-globin enhancer clusters classify as the most highly ranked super-enhancers in primitive erythroid cells.

**Fig. 2:**
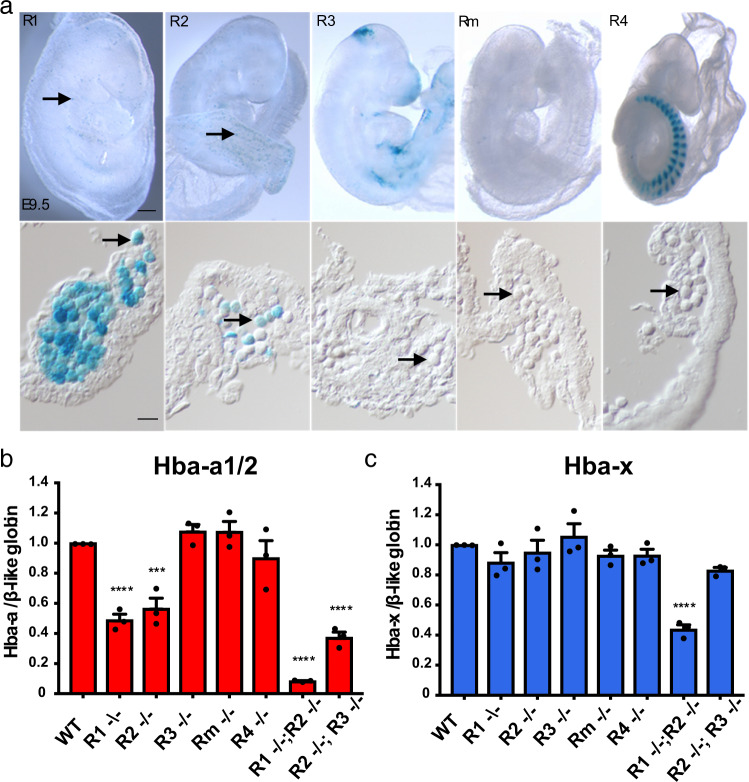
Functional analysis and contribution of each enhancer in primitive erythroblasts. **a** Representative LacZ-stained transgenic E9.5 embryo for each of the enhancer constructs (upper) and sections through the yolk sac with a population of hematopoietic cells (lower). R1 and R2 show activity in the majority of erythroid cells; there is no detectable activity in these cells from R3, R4, or the Rm element. Arrows indicate yolk sac blood islands in the upper panels and individual erythroblasts in the lower panels. Scale bar represents 250 μm in the upper panels and 25 μm in the lower panels. Numbers of positively stained embryos are summarized in Supplementary Table 2. **b**, **c** NanoString quantification of the ratio of *Hba-a1/2* (**b**) and *Hba-x* (**c**) to β-like globin transcripts (comprising *Hbb-b1, Hbb-bh1, Hbb-bh2, Hbb-y)* from steady-state RNA isolated from primitive erythroblasts isolated from homozygote mice at E10.5; mutant samples are normalized to wild-type. *N* = 3 biologically independent samples. Data are presented as mean ± SEM. A significant reduction in the α-globin:β-globin-like transcript ratio is observed in R1^−/−^, R2^−/−^ and the R2^−/−^; R3^−/−^ double-knockout embryos in comparison to wild-type. A 90% reduction in the ratio is seen in the R1^−/−^; R2^−/−^ double-knockout embryos. The ζ-globin: β-globin-like transcript ratio is only significantly reduced (by 60%) in the R1^−/−^; R2^−/−^ double-knockout embryos. *** denotes *p* ≤ 0.001; **** denotes *p* ≤ 0.0001; statistical significance was calculated using a one-way ANOVA with Dunnett correction for multiple comparisons. Source data are provided as a Source Data file.

Next, each enhancer element in the α-globin cluster was evaluated in primitive erythroid cells using a mouse transgenic system^26,27^. A vector containing a candidate enhancer, a minimal promoter and the LacZ gene were integrated into chromatin at random positions in the mouse genome. β-Galactosidase staining of embryos shows only the R1 and R2 elements exhibit positive activity in hematopoietic cells at E9.5, with 9/9 and 4/4 LacZ-stained embryos exhibiting expression revealing erythroid enhancer activity (Fig. 2a, Supplementary Table 2). Tissue sections from LacZ positive mice confirmed strong enhancer activity for R1 and R2 with no detectable activity for the remaining three elements (R3, R4 and Rm).

To investigate the role of each enhancer in vivo we utilized mouse models in which all five components of the α-globin enhancer cluster have been knocked out individually and in informative combinations^12^. Quantitation of globin mRNA in primitive erythroid cells, using Nanostring methodology which avoids amplification bias^28^, shows that the R1 and R2 elements together are responsible for ~90% of α-globin expression in primitive erythroid cells (Fig. 2b) with a reduction to ~50% and ~60% of normal levels in the R1^−/−^ and R2^−/−^ knockout models, respectively (Fig. 2b). Deletion of R1 appears to have a greater effect on α-globin expression in primitive cells than in definitive, although this is not statistically significant. The overall effects of these enhancer knockouts are similar to those seen in definitive erythropoiesis although R1 seems more active than R2 in primitive cells while the reverse is true in definitive cells. As in definitive cells^12^, the R1/R2 homozygous double knockout in primitive cells again demonstrates a simple additive effect of the enhancers on α-globin expression.

The effects of these enhancer knockouts on ζ-globin expression are different. Knockout of each enhancer element individually has no detectable effect on ζ-globin expression; however, homozygous knockout of R1 and R2 together led to a significant reduction in ζ-globin expression (Fig. 2c). This finding agrees with our transgenic assay for enhancer activity, with only R1 and R2 showing in vivo activity. Importantly, we noted that ζ-globin is much more robust to the simultaneous R1/R2 double knockout, as it suffers only a ~50% reduction in expression compared to the 90% reduction of α-globin expression in the same knockout. It thus appears that there is redundancy between the individual enhancer elements making up the α-globin super-enhancer when considering their effects on ζ-globin expression in contrast to their effects on α-globin. One plausible explanation for this is the extended ζ-globin promoter (~400 bp) which is highly conserved throughout evolution^11^. DNAseI digital footprinting suggests the ζ-promoter is capable of recruiting activating *trans*-acting factors directly, thereby rendering ζ-globin less reliant on any single enhancer element (Supplementary Fig. 2).

In summary, the expression of the ζ-globin gene is regulated by the same set of enhancers as the α-globin genes, particularly R1 and R2. Τhere is no evidence for embryonic-specific enhancer activity. Therefore, the developmental specificity of ζ-globin expression appears to lie within the ζ-globin gene and its flanking regions.

### ζ-globin lies within a region of facultative heterochromatin

**Fig. 3:**
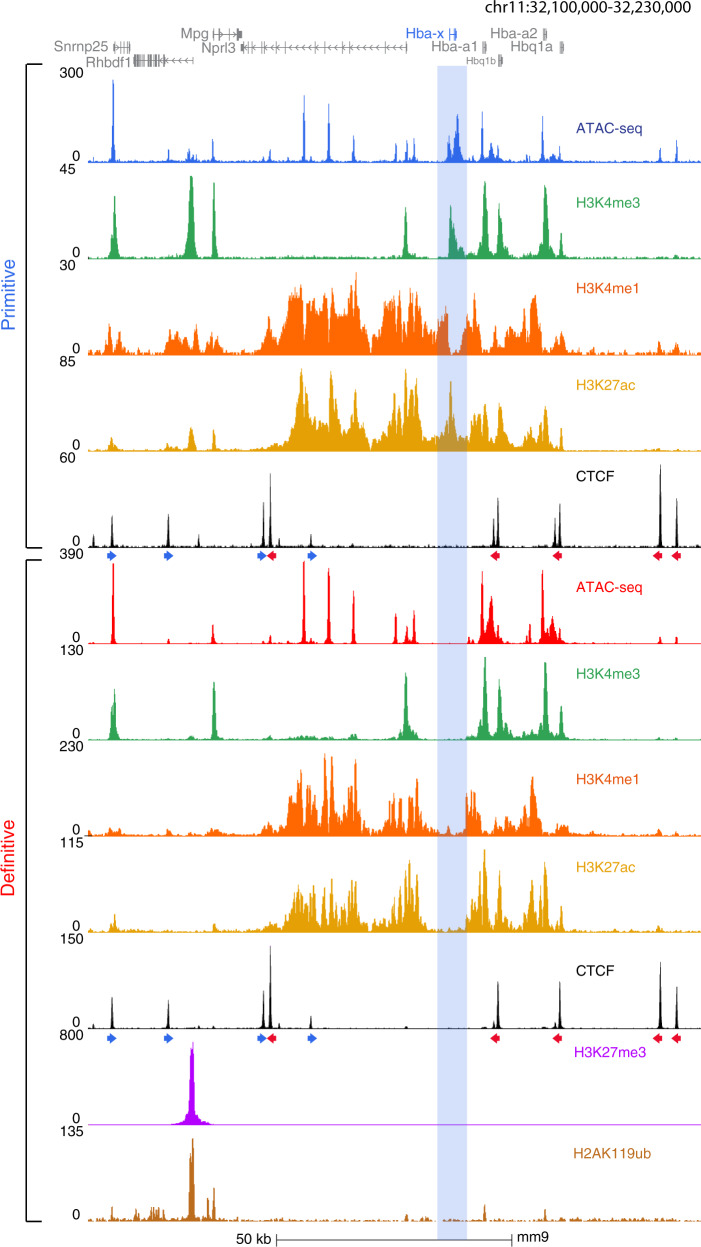
The ζ-globin gene lies within a small, discrete region of hypoacetylated chromatin in definitive erythroid cells. ChIP-seq tracks for H3K4me3, H3K4me1, H3K27ac, and CTCF in primitive erythroid cells at the α-globin locus. An ATAC-seq track is shown for orientation. The *Hba-x* gene is highlighted in blue throughout. The orientation of CTCF sites is shown below the CTCF tracks. The same histone marks for definitive erythroid cells are shown, along with H3K27me3 and H2AK119ub which are associated with polycomb repression.

These findings raised the question of how the ζ-gene remains silenced in definitive erythroid cells despite its close proximity to its cognate super-enhancer, and in the presence of transcription factors (e.g. Gata1) which bind the gene in primitive cells. To determine the silencing mechanism, we characterized the chromatin profile across the entire α-globin cluster and its flanking regions in primitive and definitive erythroid cells (Fig. 3).

We performed ChIP-seq for histone modifications associated with enhancers (H3K4me1) and promoters (H3K4me3). This showed that in primitive cells the ζ-promoter appears as an ATAC accessible region with H3K4me3 and H3K27ac modifications, indicating an active promoter (Fig. 3). By contrast, in definitive cells, the promoter is ATAC inaccessible and inactive (H3K4me3 and H3K27ac negative). A region of hypoacetylated chromatin extends ~10 kb across the entire ζ-gene and its flanking regions in definitive cells (Fig. 3). We were unable to identify other potential silencing modalities affecting the ζ-gene in definitive cells including the formation of heterochromatin (H3K9me2 and H3K9me3), DNA methylation, and/or Polycomb repression (PRC2:H3K27me3 and PRC1: H2AK119Ub) (Fig. 3 and Supplementary Fig. 5). We also examined the distribution of CTCF boundary elements, but these appeared identical in primitive and definitive cells (Fig. 3).

It has previously been shown that histone de-acetylase (HDAC) inhibitors may de-repress ζ-globin expression in mouse erythroid cells^29^. We, therefore, examined the effect of an HDAC inhibitor on mouse erythroid cells in which the coding sequence of the ζ-globin gene has been replaced with sequence encoding mVenus, allowing a sensitive read-out of transcription. HC toxin, a known HDAC inhibitor^29^, was added to heterozygote ζ-Venus erythroid cells for 48 h and ζ-globin expression measured (via YFP signal). We found an increase of ζ-globin expression in a dose-dependent fashion such that ~16% of cells were positive when treated with 16 nM of HC toxin (Supplementary Fig. 6) confirming that histone deacetylation plays a role in ζ-globin repression. In summary, the ζ-globin gene in definitive cells appears to lie in a discrete region of inaccessible, hypoacetylated facultative heterochromatin which contributes to its silenced state in these cells.

### Absence of ζ-globin promoter/enhancer contact

**Fig. 4:**
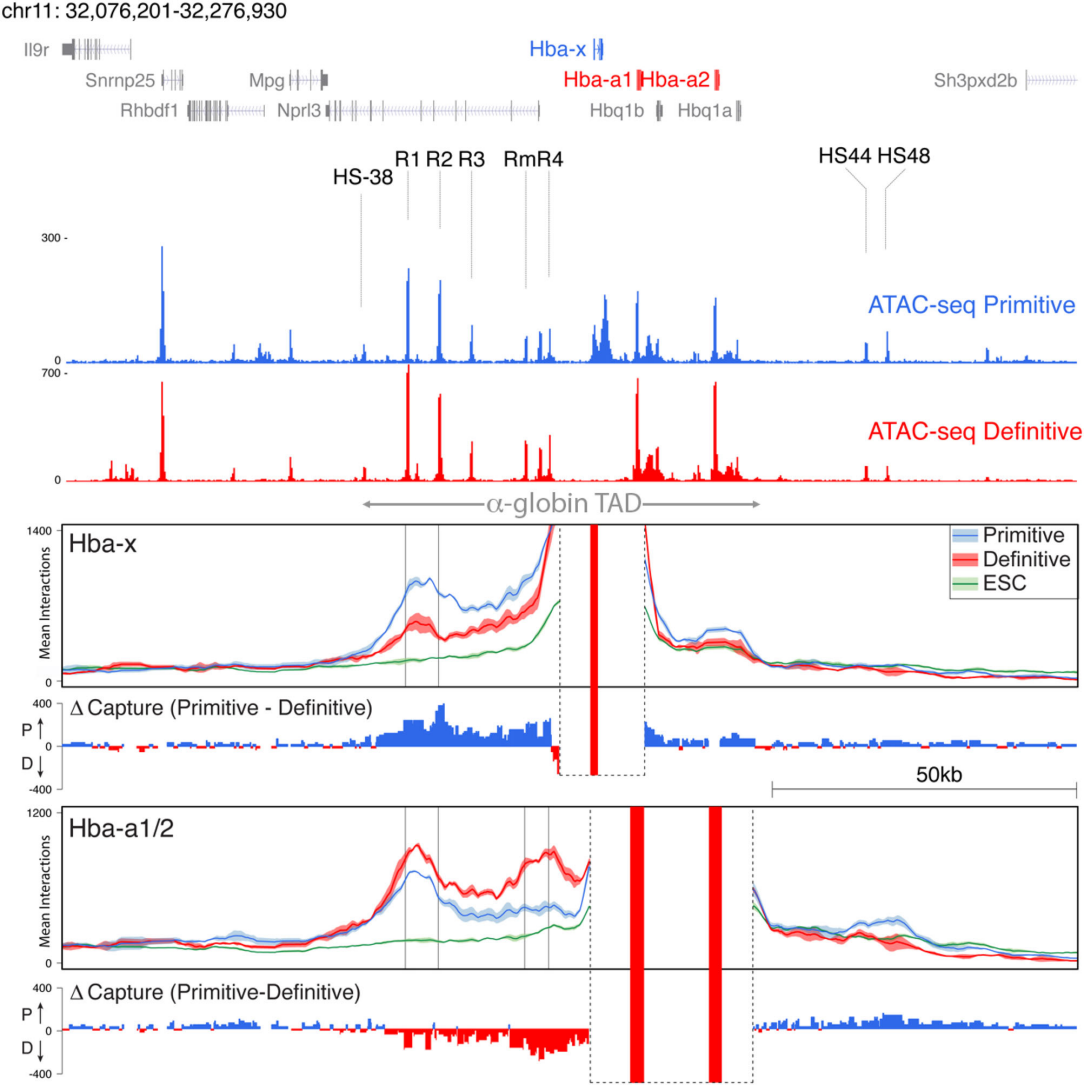
The ζ-globin gene remains in proximity to the α-globin superenhancer in definitive erythropoiesis. Normalized Capture-C data from the viewpoint of the *Hba-x* promoter and the *Hba-a1/2* promoter (lower panels), ATAC-seq tracks are shown above for orientation. The mean, plus and minus one standard deviation (s.d.), of sliding 5 kb windows are visualized. Differential tracks (ΔCapture-C) show a subtraction (Primitive−Definitive) of the mean number of meaningful interactions per restriction fragment. Red vertical bars indicate the position of viewpoints; an ATAC-seq track from primitive erythroid cells is shown with the enhancer elements, promoters, and CTCF boundary elements denoted. Data from three independent experiments are merged. The extent of the alpha-globin TAD is indicated with a double-headed arrow.

We have previously shown that the mouse α-globin cluster is contained within a topologically associating domain (TAD) (Fig. 1). To determine whether the ~65 kb ζ-α-globin sub-TAD also forms in primitive erythroid cells, we performed next-generation Capture-C^30^ from the viewpoints of the ζ-globin promoter, the α-globin promoters, the R1 and R2 enhancer elements, and the CTCF boundary element (HS-38) that defines the proximal edge of the compartment (Fig. 4 and Supplementary Fig. 7). In primitive cells, the self-interacting domain is delimited by the same boundaries as in definitive cells and binding of the architectural protein CTCF occurs at the same sites in both cell lineages. We found increased interactions between the enhancer elements and ζ-globin in primitive cells compared to definitive cells (Fig. 4). This suggests that during primitive erythropoiesis ζ-globin expression is mediated, at least in part, via proximity between the enhancers and the ζ-globin promoter consistent with our observations on removing these enhancers in primitive cells.

**Fig. 5:**
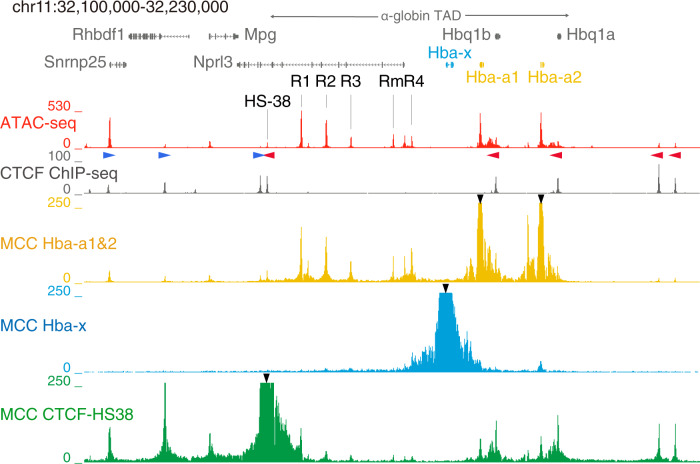
Micro Capture-C (MCC) shows that the ζ-globin gene does not contact the α-globin enhancers in definitive eryrthroblasts. MCC from the α-globin promoters (black arrowheads) shows specific contact of these genes with the enhancers, interactions are represented by peaks at the enhancer elements in this track. Conversely, MCC from the viewpoint of the *Hba-x* promoter (black arrowhead) shows *Hba-x* does not contact the α-globin enhancers, or indeed any other sites within the α-globin TAD, suggesting it is isolated. MCC from the CTCF-binding site at HS-38 (black arrowhead) confirms this as it shows interaction with the α-globin genes but no contact with *Hba-x*. ATAC and CTCF binding profiles are shown for orientation. The orientation of CTCF-binding sites is shown by red and blue arrows and the extent of the α-globin TAD is indicated by a double-headed arrow.

Although chromatin interactions between the ζ-globin gene and the region containing the major R1 and R2 enhancers decrease in definitive cells, these interactions remain enriched compared to non-erythroid mESCs (Fig. 4 and Supplementary Fig. 7). Since such interactions are thought to play an important role in the activation of gene expression, the close proximity of strong enhancers and the ζ-promoter is unexpected as the embryonic ζ-gene remains silent in definitive cells. It is possible that the relatively broad, weak interactions detected by Capture-C between the ζ-promoter and the enhancers reflect the larger chromatin structure of this region in definitive cells rather than specific interactions between the elements themselves. To examine this at higher resolution, we analyzed the interactions between the enhancers and the ζ-promoter in definitive erythroid cells using a new chromatin conformation capture (3C) method termed Micro Capture-C (MCC), which allows physical contacts to be determined at base-pair resolution^31^ MCC was able to detect a strong interaction between the α-globin promoters and their enhancers in definitive cells. Remarkably, we find no such interaction between the enhancers and the ζ-globin promoter in definitive cells, despite their proximity as identified by Capture-C (Fig. 5).

### Identifying factors involved in ζ-globin silencing

ζ-globin is normally undetectable in definitive human erythroid cells, although has rarely been found to be upregulated by cis- or *trans*-acting mutations. It is detectable at levels >1% in αα/–^SEA^ individuals, who carry a common deletion of the α-globin genes^32,33^. Of interest ζ-globin is also elevated in previously reported individuals trisomic for chromosome 13 (Patau syndrome) and this is thought to occur via decreased levels of the transcription factor MYB as a result of increased expression of two microRNAs (mIR-15a and miR-16-1)^34^. Patients who are compound heterozygotes for mutations in *KLF1* consistently have elevated levels of ζ-globin expression^35,36^. Several previous reports have also shown incidentally that ζ-globin expression in mouse and/or human may be upregulated when particular transcription factors and co-factors are perturbed. These include POGZ^37^, BCL11A^38^, SOX6^39^, LRF^40^, and KLF3/8^41^. It has also been shown that several of these proteins play a key role in the developmental silencing of fetal γ-gobin expression^37,38,40,42^. Of interest, amongst other complexes, all of these proteins converge on the nucleosome remodeling deacetylase (NuRD) multiprotein complex^15,40,43^. This contains HDAC1 and HDAC2 which are antagonized by HC toxin^29^ and Supplementary Fig. 6.

Here we investigated in detail the two most intensively studied silencers of globin gene expression; LRF and BCL11A. To study the knockout of *Lrf* we crossed heterozygous mice in which Exon 2 of the *Lrf* gene had been removed, abrogating *Lrf* expression^40,44,45^ and analyzed cells taken at E12.5–E13.5 from primary fetal liver. Knockout of *Lrf* causes apoptosis and a differentiation block in erythropoiesis^46^, therefore single-cell expression was performed on cells sorted by flow cytometry to ensure that differentiation was matched between WT, heterozygous and homozygous knockout cells. Alongside globin expression, we also analyzed the expression of genes known to change during erythroid differentiation (Supplementary Table 3). Principal component analysis (PCA) demonstrated clustering the cells by differentiation stage, rather than by genotype, demonstrating that cells were appropriately and equally stage-matched (Supplementary Fig. 8). ATAC-seq was also performed on sorted cells.

**Fig. 6:**
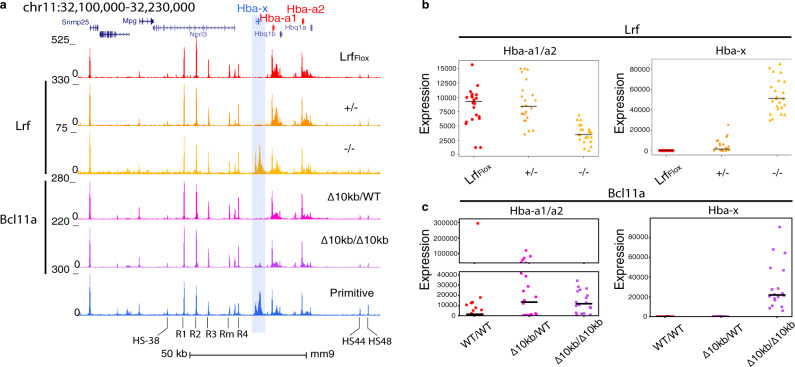
Lrf and Bcl11a play a role in silencing ζ-globin expression in mice. **a** ATAC-seq at the α-globin locus in *Lrf*^Flox^/*Lrf*^Flox^ (*Lrf*^Flox^), *Lrf*^Flox/-^ (+/−), *Lrf*^−/−^ (−/−) erythroid cells, *Bcl11a*^Δ10kb/WT^(Δ10kb/WT) and *Bcl11a*^Δ10kb/Δ10kb^ (Δ10kb/Δ10kb) erythroid cells were taken at E12.5–E13.5. The ζ-globin gene is highlighted in blue, tracks from definitive and primitive erythroid cells (E12.5 and E10.5 respectively) are also shown. **b**, **c** Single-cell expression of α- and ζ-globin when *Lrf* and *Bcl11a* expression have been perturbed. Data are generated from littermates. Each dot represents a single cell, and the median expression is indicated by a horizontal line.

When Lrf is removed, *Hba-x* expression is markedly de-repressed at E12.5 with a modest reduction in adult α-globin expression, and chromatin accessibility at the *Hba-x* gene resembles that seen in primitive erythropoiesis (Fig. 6). To ensure that there were no contaminating primitive erythroid cells we also examined globin expression and chromatin accessibility at the β-globin locus (Supplementary Fig. 8). Importantly this showed no change in chromatin accessibility or expression of the *Hbb-y* gene, consistent with previous reports that *Lrf* knockout de-represses *Hbb-bh1*, but has no effect on *Hbb-y*^40^.

To investigate the effects of Bcl11a on ζ-globin silencing, we utilized a mouse model harboring a deletion of a 10 kb region comprising two erythroid-specific enhancers for *Bcl11a*^47^. This causes a reduction of *Bcl11a* expression in erythroid cells without affecting levels in other tissues. We confirmed homozygous deletion of the composite enhancer leads to a decrease in *Bcl11a* equivalent to that found in primitive erythropoiesis (Supplementary Fig. 9). Since a reduction in *Bcl11a* expression de-represses both embryonic globins at the β-globin locus, we performed single-cell expression for genes known to vary between primitive and definitive erythropoiesis (Supplementary Table 4) as well as globin expression. PCA showed WT, heterozygous and homozygous cells cluster together and absent embryonic β-like globin expression in WT cells confirming that definitive erythroid cells had been analyzed (Supplementary Fig. 9). Removal of the *Bcl11a* composite enhancer leads to ζ-globin de-repression and changes in chromatin accessibility, as assessed by ATAC-seq. The effects are, however, less than those seen for knockout of *Lrf* (Fig. 6), one possible explanation for this difference may be because of the very small residual *Bcl11a* expression in the absence of the enhancer (Supplementary Fig. 9).

These results provide evidence that *Lrf* and *Bcl11a* are involved in the repression of ζ-globin in fetal definitive erythroid cells and most likely exert their effects via chromatin remodeling and histone deacetylation.

### LRF and BCL11A play a role in ζ-globin silencing in humans

To determine whether LRF and BCL11A also play a role in ζ-globin repression in humans, we turned to the HUDEP-2 cell line^48^. This definitive erythroid cell line undergoes terminal erythroid differentiation upon removal of doxycycline and expresses predominantly adult hemoglobins with a very low background of embryonic and fetal globin expression^48^ (Supplementary Fig. 10). Chromatin accessibility, as assessed by ATAC-seq, histone modifications and CTCF binding is very similar between differentiated HUDEP-2 cells and human CD34^+^ hematopoietic stem and progenitor cell (HSPC)-derived primary erythroblasts at the α-globin locus (Supplementary Fig. 11). Here, we generated clones in which LRF and BCL11A were knocked out individually and together.

**Fig. 7:**
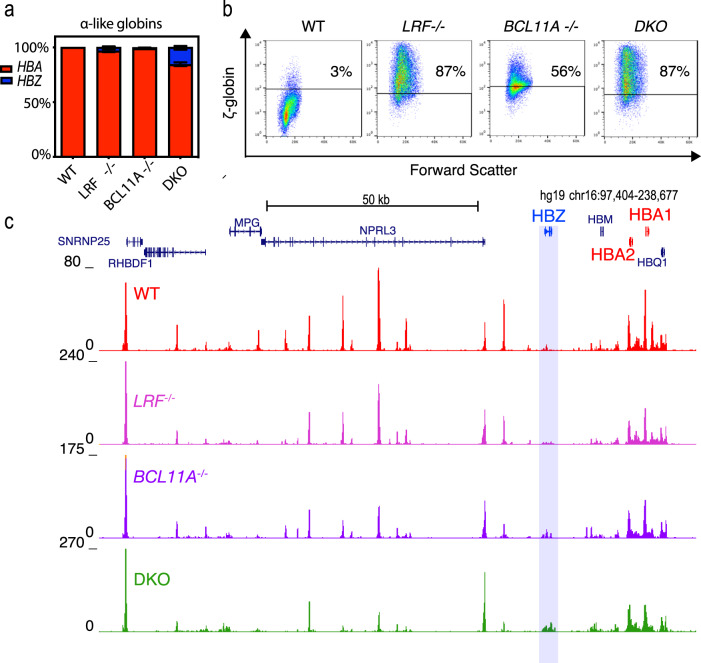
Role of LRF and BCL11A in humans. **a** Bargraphs showing the proportion of ζ-globin transcripts (*HBZ*) to adult α-globin at Day 10 of differentiation (bars show the mean ± SD); *N* = 3 biologically independent samples). **b** Flow cytometry for the ζ-globin chain at Day 10 of differentiation. The proportion of ζ-globin positive cells are shown. The negative line was set using an isotype control for each cell line. **c** ATAC-seq at Day 10 of differentiation on 10,000 cells. The ζ-globin (*HBZ*) gene is indicated by the blue bar. Source data are provided as a Source Data file.

Knockout of LRF and BCL11A individually led to detectable ζ-globin expression at both the RNA and protein level (Fig. 7a, b and Supplementary Fig. 12) with a small increase in histone acetylation seen over the ζ-globin gene when compared to WT HUDEP-2 cells (Supplementary Fig. 13). However, chromatin at the ζ-globin promoter remains inaccessible by ATAC-seq (Fig. 7c). Knockout of both BCL11A and LRF together had a greater effect, leading to ζ-globin expression which accounts for ~15% of the total α-like globin transcription, suggesting BCL11A and LRF act on ζ-globin via independent pathways, as they do at the β-globin locus^40^. Although chromatin accessibility and expression increase, it does not return to levels seen in primitive erythropoiesis, suggesting there are additional silencing mechanisms or that additional pathways are involved in fully activating ζ-globin expression.

To determine whether LRF and BCL11A exert their effects by binding directly to the ζ-globin promoter, we re-analyzed previously published ChIP-seq and CUT&RUN data-sets for LRF and BCL11A in HUDEP-2 cells^40,49,50^. Both show direct binding to the ζ-globin promoter and consensus sequences for BCL11A and LRF are present in both the human and mouse ζ-globin gene promoters (Supplementary Fig. 5). This suggests that both transcription factors exert their effects by binding directly to the ζ-globin promoter in a similar fashion to their binding at the γ-globin promoter in the β-globin locus^49,50^. There are consensus binding sequences for LRF and two sites for BCL11A in the *HBZ* promoter, however, there is only a single predicted Bcl11a-binding site in the *Hba-x* promoter. Further work will be required to examine the mode of action of these repressors in human and mouse erythroblasts.

## Discussion

Here, we have demonstrated that the α-globin locus in primitive erythroid cells is contained within the same ~65 kb self-interacting chromosomal domain (sub-TAD) as that found in definitive erythroid cells. All of the enhancers present in definitive erythroid cells are also active in primitive erythroid cells and collectively they classify as a super-enhancer. The ζ-gene is less dependent on these enhancers than the α-genes for full activity. Whereas the enhancers behave in an additive manner with respect to α-globin expression in embryonic and definitive erythroid cells, they appear to act redundantly on ζ-gene expression. Finally, the same CTCF sites form the boundary elements in both embryonic and definitive erythroid cells; there are no embryonic-specific CTCF insulators responsible for reconfiguring the locus or silencing ζ-globin expression. Together, these findings show the ζ-globin gene is encapsulated in a very similar chromatin environment within the α-globin cluster during development.

These findings are consistent with previous observations of randomly integrated transgenes in mouse models which concluded that silencing of the embryonic ζ-globin gene is mediated by sequences lying within or closely flanking this gene^22^. Here we have investigated the mechanism by which the ζ-gene is silenced. In primitive erythropoiesis, when both the ζ- and α-genes are co-expressed, there is a broad region of acetylation across the entire ~65 kb self-interacting domain. By contrast, in definitive erythropoiesis, the ζ-gene, lying at the center of this ~65 kb domain, is packaged into an extended 10 kb region of what appears to be facultative heterochromatin marked by histone de-acetylation (Fig. 3). This includes the entire ζ-gene and its flanking regions. Importantly, treatment of definitive cells with an inhibitor (HC toxin) of specific histone de-acetylases (HDAC1 and HDAC2) partially de-represses ζ-globin expression in adult cells suggesting that silencing is normally maintained, to some extent, by histone de-acetylation of this region of chromatin.

This presents an unusual situation in which the embryonic genes, which we have shown to respond to the α-globin enhancers and their associated transcription factors in embryonic life, are silenced despite lying within 7 kb of an erythroid-specific super-enhancer. The α-globin genes lying a further 7 and 20 kb away on the chromosome are, at the same time, fully activated.

**Fig. 8:**
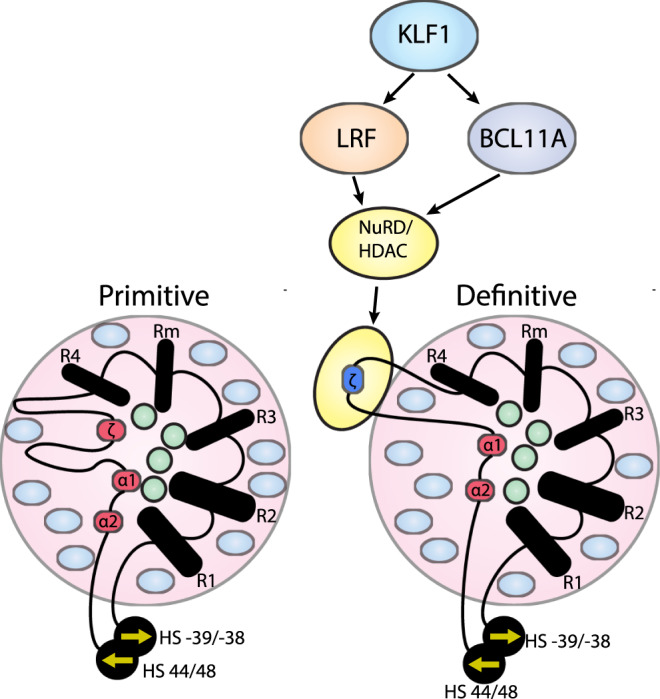
Model of ζ-globin activation and repression. LRF and BCL11A both interact via the NuRD complex leading to ζ-globin hypoacetylation and silencing in definitive (adult) erythroid cells and are regulated upstream by KLF1^15^. Pink circles represent the domain of H3K27 acetylation, green circles, and blue ovals represent transcriptional activators and repressors respectively. The yellow oval indicates a separate compartment containing the hypoacetylated region containing the ζ-globin gene. HS-39/HS-38 and HS44/48 refer to the CTCF sites known to be involved in the formation of the α-globin domain^19^ and the arrows indicate directionality, see main text.

We have previously shown the α-globin genes interact with all elements of the super-enhancer in a regulatory hub structure when active in definitive erythroid cells^51^. To investigate how the ζ-globin promoter interacts with the enhancers in primitive and definitive cells, we performed various 3C assays. Capture-C analysis revealed that the self-interacting domain formed in primitive erythroid cells appears to be broadly similar to that found in definitive erythroid cells. However, contacts between the enhancers and the ζ-globin gene promoters are more pronounced in primitive cells. Of interest, Capture-C shows the silent ζ-gene still appears to be in relatively close 3-D proximity to the enhancers in definitive cells (Fig. 4). However, when we examined this using MCC, which has a much higher resolution, we found that despite their proximity there are no specific interactions between the enhancers and the silent ζ-globin promoter in definitive cells (Fig. 5). This indicates that the ζ-globin promoter is excluded from the hub in definitive cells (Fig. 8). Interestingly, a similar, but much more striking conformational change, is observed between fetal and adult stage human erythroid cells at the β-globin locus, where the *BGLT3*, *HBBP1*, *HBE,* and *HBG1/2* genes are sequestered away from the active transcriptional hub in adult stage erythroid cells^52^.

Recent interpretations of enhancer/promoter interactions and their functional significance suggest that enhancers may establish a phase-separated compartment. Some have suggested that such transcriptional compartments form via liquid–liquid phase-separation engendered by multiple low-affinity protein interactions between intrinsically disordered domains^53,54^. Such compartments contain a high concentration of transcription factors and co-factors, including mediators, to facilitate active transcription. Interestingly, there is an excess of transcriptional repressors in erythroblast nuclei^55^, suggesting close contact with enhancers may bring promoters into proximity with the transcriptional activators known to bind there. It is thought that spatially separated clusters may be relatively small and this has been termed microphase separation^56^. One mechanism underlying separation may be the behavior of chromatin as a liquid or solid, depending on post-translational histone modifications, particularly acetylation^57^. In terms of scale, we have previously shown that the 65 kb alpha-globin TAD forms a domain of ~500 nm in diameter in definitive erythroblasts^58^. Studies in vitro and in vivo suggest that condensates of 100 nm in diameter form^57,59^. Therefore, it is plausible that the 10 kb hypoacetylated domain surrounding the zeta-globin gene seen here may form a functionally distinct condensate. The exclusion of a condensed hypoacetylated domain from an otherwise hyper-acetylated transcriptional compartment could explain how the ζ-gene is excluded from activation, despite its physical proximity and despite the presence of known transcriptional activators of the ζ-globin gene, such as Gata1. However, further work, including single-cell imaging studies, will be required to test this hypothesis.

How are ζ-gene and its flanking regions recognized and de-acetylated in definitive erythroid cells? HDACs do not bind DNA directly, rather, they associate with multiprotein complexes including transcription factors that can be recruited to specific DNA or RNA sequences. BCL11A and LRF, two previously described transcription factors responsible for repressing globin gene expression, both associate with the NuRD complex and may thereby recruit HDAC proteins to specific globin genes^40,60^. Previous work has suggested that these proteins are recruited to the ζ-gene in mouse^40^ and human^49,50^ (Fig. 8). Given this, and the localized histone deacetylation seen over the ζ-globin gene associated with silencing, we further investigated the effects of *Bcl11a* and *Lrf* knockout on ζ-globin expression and the chromatin landscape at the α-globin locus in mice. We found that removal of either transcription factor in vivo in definitive murine erythroid cells leads to some degree of ζ-globin de-repression and increased chromatin accessibility. Similar effects were observed in a human definitive erythroid cell model, although knockout of both transcription factors does not lead to full restoration of ζ-globin expression to the levels seen during primitive erythropoiesis. This suggests either that there may be other redundant pathways contributing to the ζ-globin silencing or that critical positive regulators of ζ-globin expression are not present in definitive cells (Fig. 8). Both possibilities are being investigated.

Although this work addresses the fundamental question of how genes are silenced in development it is also of biomedical importance. A common lethal form of α-thalassemia (the Bart’s Hydrops fetalis syndrome: BHFS) occurs due to inheritance of two alleles in which both α-globin genes have been deleted in *ci*s (ζ-^SEA^/ζ-^SEA^)^15^. The most common allele (ζ-^SEA^) occurs at a high frequency throughout South-East Asia^61^ in some regions up to 1:200 conceived infants have this condition^62^. Most often an affected fetus dies in the second or third trimester of pregnancy^63^. However, advances in fetal medicine and neonatal care mean that a small number of affected children have survived with this condition but require lifelong blood transfusion^63^. Reactivation of the intact but silenced ζ-globin gene would be a curative therapy in such cases, and mouse models show that this would be a viable strategy^64^.

The majority of data presented here relates to the mouse ζ-globin locus, there are several similarities between the mouse and human loci, including conservation in sequence and the positioning and order of the elements^11^ and temporal similarities in the silencing of the ζ-globin gene; mice where the human α-globin locus has been substituted in place of the mouse α-globin locus also have normal human ζ-globin expression and silencing^17^. The most prominent difference between the mouse and human α-globin loci is the absence of large CpG islands^11^. However, the CpG island in the human ζ-globin gene is located at the 3′ region and its methylation inversely correlates with transcription (Supplementary Fig. 5) suggesting that it does not play a clear role in human ζ-globin silencing. The work here thus provides therapeutic targets for ζ-globin de-repression and suggests that many of the current strategies being developed to treat the β-haemoglobinopathies^3,65^ may also be of value in severe α-thalassemia. Overall, our understanding of the developmental regulation of the globin genes will underpin general strategies to re-activate silenced embryonic and fetal genes that may rescue phenotypes that are caused by mutations in the adult paralogues of these genes.

## Methods

### Mice

All mouse work was performed in accordance with UK Home Office regulations under project license numbers 30/3328 and PP1728514 with ethical review from the University of Oxford Medical Sciences Division Local Ethical Review Panel. Mouse models where each of the regulatory elements (R1, R2, R3, Rm, and R4) have been deleted individually and in informative combinations have been previously reported^12^. Wild-type cells were obtained from C57Bl/6 mice.

A breeding pair of Lrf^Flox/Flox^ mice were a kind gift from Pier Pandolfi^40^. These were crossed with WT to obtain heterozygotes which were then crossed with a mouse-like expressing Cre under the control of the *Gata1* regulatory elements^66^, in which Cre is expressed in the germline and thus affects efficient germ-line excision of floxed regions. *Lrf*^Flox/−^ mice were interbred to produce litters with a combination of *Lrf*^Flox/Flox^ (WT), *Lrf*^*Flox/-*^(+/−), and *Lrf* ^−*/−*^ (−/−) litters.

A mouse with the composite enhancer block for Bcl11a (corresponding to 10 kb) deleted (*Bcl11a*^Δ10kb^)^47^ was imported as a kind gift from Professor Stuart Orkin.

### Mouse embryonic stem cells

E14 mouse ES cells (derived in house) were maintained in GMEM (Life Technologies) supplemented with: FBS (10%, Gibco), sodium pyruvate (1 mM, Life Technologies), non-essential amino acids (1 mM, Life Technologies), Glutamine (2 mM, Gibco), Penicillin/Streptomycin (P/S; 100 IU/ml, Gibco), 1000 IU/ml Leukemia Inhibitory Factor (LIF, Millipore) and 0.1 mM β-Mercaptoethanol (Sigma-Aldrich) at 37 °C with 5% CO_2_ incubation on gelatin (0.1%, Sigma-Aldrich)-coated flasks.

### Primary primitive erythroblasts

Primary primitive erythroblasts were isolated from yolk sacs at E10.5. Because of the semi-synchronous nature of primitive erythroid maturation, these primary primitive erythroid cells were highly homogeneous at the morphological stage of intermediate erythroblasts. Morphological examination of cells for each experiment was undertaken to ensure homogeneity.

To ensure that cells in which the ζ-globin gene is maximally transcribed were analyzed, primitive erythroblasts were isolated from mouse embryonic blood at E10.5, E11.5, and E12.5, and ζ-globin mRNA levels measured by qPCR. In agreement with previous studies^14^, in E10.5 erythroblasts ζ-globin accounted for 40% of the transcriptional output from the α-globin locus. A similar level of ζ-globin RNA was found at E11.5, reducing to ~20% by E12.5 (Supplementary Fig. 1). Since globin RNA has a relatively long half-life it is possible that nascent transcription from ζ-globin may already be repressed at E11.5. We, therefore, selected primitive erythroblasts at E10.5 to ensure that ζ-globin expression would be maximal. Because primitive erythroblasts mature in a semi-synchronous wave^5^ all primitive cells were at the intermediate/polychromatic stage when analyzed (Supplementary Fig. 1). For comparison, definitive erythroid cells were obtained from fetal liver culture (derived from E12.5 embryos) and synchronized by Ter119 depletion, followed by fluorescent activated cell sorting (FACS) using CD44 prior to differentiation (Supplementary Fig. 1). The developmental staging was confirmed by FACs analysis for Ter-119 and CD71 and morphology, to ensure that all cells were at the intermediate stage (see the “Methods” section and Supplementary Fig. 1).

### Definitive erythroid cells

Definitive erythroid cells were obtained from a well-established fetal liver culture system^67^. Fetal livers were dissected from E12.5 mouse embryos, and the dissociated cell suspension was expanded for 5–7 days in Stempro (Invitrogen) supplemented with Epo (1 U/ml), SCF (50 ng/ml), and dexamethasone (1 μM). After the expansion of the cultures, mature red blood cells were removed by negative selection for Ter119 using anti-Ter119 magnetic beads (Miltenyi Biotec), to obtain a population of erythroid precursors, which was then purified by cell sorting to isolate CD44 positive cells, which were sorted into expansion media and allowed to recover for 6 h. Cultures were switched to medium containing a high Epo concentration (5 U/ml) to induce differentiation into mature erythroid cells and harvested after 24 h. Differentiation was confirmed by flow cytometry analysis for Ter119 and CD71, and morphological examination of cytospins.

For MCC experiments, definitive erythroid cells were obtained from phenylhydrazine-treated mice^68,69^. Spleens were mechanically disrupted to a single cell suspension in ice-cold PBS. To isolate late-stage erythroid cells, cells from a single spleen were resuspended in 5 ml of cold PBS/10% FCS and stained with 50 μl PE anti-Ter119 antibody (RUO, BD Biosciences) at 4 °C for 30 min in the dark. After washing with 40 ml of ice-cold PBS/10% FCS, cells were resuspended in 800 μl MACS buffer (PBS, 2 mM EDTA, 0.5% BSA) and 200 μl of anti-PE magnetic beads (Miltenyi Biotec) and incubated for 20 min at 4 °C in the dark. Ter119 positive cells were isolated via auto-magnetic-activated cell sorting (MACS) and then processed for downstream applications. Purity of cells was confirmed by flow cytometry.

### HUDEP-2 cell culture

WT HUDEP-2 cells, *LRF*^−/−^, *BCL11A*^−/−^ and *DKO* cells were a gift from Dan Bauer^40^; all clones had previously been confirmed as knockout by Western Blot by the donating laboratory. Cells were maintained in the expansion phase using StemSpan SFEM medium (Stem Cell Technologies) supplemented with hSCF (50 ng/ml, Peprotech), erythropoietin (3 IU/ml, OUH hospital pharmacy), dexamethasone (10^−6^ M, OUH Hospital pharmacy), glutamine (2 mM, Life Technologies), Penicillin/Streptomycin (P/S, 100 IU/ml, Life Technologies), and doxycycline (2 μg/ml, Sigma-Aldrich) at 37 °C with 5% CO_2_ incubation. Cell concentration was maintained between 0.5 and 1 × 10^6^ cells/ml. The media was changed every 48 h whilst cells were in expansion. Cells were kept for a maximum of 3 weeks.

To induce differentiation, cells were transferred to IMDM (Life Technologies) supplemented with: Solvent/Detergent pooled AB plasma (2.5%, NHSBT), hSCF (50 ng/μl), Epo (3IU/ml), holotransferrin (330 μg/ml, Sigma-Aldrich), insulin (10 μg/ml, Sigma-Aldrich), heparin (2 IU/ml, JR hospital pharmacy), P/S (100 IU/ml), glutamine (2 mM), and doxycycline (2 μg/ml) at 37 °C with 5% CO_2_ incubation. Media was changed every 48 h. Cell concentration was maintained between 0.5 and 1 × 10^6^ cells/ml. After 7 days, doxycycline was removed from the media, and cells were then cultured for a further 3 days prior to experiments. Adequate differentiation was confirmed using flow cytometry and cytospin.

### Flow cytometry of HUDEP-2 cells

All cells were analyzed by flow cytometry using the Attune NxT instrument and proprietary software (Thermo Fisher). Data were analyzed using flowJo version 10.3.

After washing HUDEP-2 cells twice with FACS staining buffer (PBS, 10% FCS, Life technologies), cells were resuspended in 100 μl of FACS buffer and stained with the following antibodies at 4 °C for 30 min in the dark: PE mouse anti-human CD235a (GA-R2, BD Biosciences 1:100), FITC mouse anti-human Band 3 (BRIC 6, NHSBT 1:50), APC mouse anti-human CD49d (9F10, BD Biosciences 1:40), PE/Cy7 CD34 (BioLegend 1:100), APC/Cy7 CD36 (BioLegend 1:100), PerCP/Cy5.5 CD71 (BioLegend 1:100). Antibodies were titrated to obtain the optimum signal. Cells were washed twice with FACS buffer, then resuspended in FACS buffer containing 1 ng/μl of Hoechst 33342 (Invitrogen). Negative, single-color controls and fluorescent minus one (FMO) samples were prepared for calibration.

Intracellular staining of HUDEP-2 cells was performed using the Intracellular Fixation & Permeabilization Buffer Set (eBioscience) in accordance with the manufacturer’s instructions. Prior to fixation, cells were stained with the LIVE/DEAD^®^ Fixable Aqua Dead Cell Stain Kit (Life Technologies) in accordance with the manufacturer’s instructions. Hybridoma supernatant was used as the primary antibody (15 μl in 100 μl)^69^, PE goat anti-mouse IgG (SouthernBiotech) was used as the secondary antibody. A mouse IgG1 (MOPC-21 BD Biosciences, 1:200) was used as an isotype control. HbF staining was carried out with FITC mouse anti-human HbF (2D12, BD Biosciences) with a FITC mouse IgG1 Isotype control (MOPC-21, BD Biosciences).

### Hemoglobin analysis

Hemoglobin was analyzed by isoelectric focusing (IEF) (Resolve; PerkinElmer) as per manufacturer’s instructions running for 45 min at 300 V at 15 °C.

### Analysis of steady-state RNA levels in mouse models

Cells were lysed in TRI Reagent (Sigma), and RNA was extracted according to the manufacturer’s instructions. Samples were treated with DNase I using Turbo DNA-free DNase (Ambion) to remove genomic DNA. 100 ng to 1 μg of RNA was converted to cDNA using the Superscript III first-strand synthesis SuperMix (Invitrogen). Quantification of total RNA transcripts was performed using NanoString nCounter technology, employing a customized probe set (Supplementary Table 5). Data were subjected to Levene’s test to confirm the homogeneity of variance, followed by one-way ANOVA with Dunnett’s correction.

### Generation of a Hba-x reporter mouse

pSpCas9(BB)-2A-Puro (PX459) V2.0 was obtained as a gift from Dr. Feng Zhang (Broad Institute) obtained via Addgene (plasmid # 62988). A pair of 24-mer oligonucleotides (5′-CACCGGCTCAGGGGTGAAGTCGGC and 5′AAACGCCGACTTCACCCCTGAGCC) containing a gRNA sequence targeting the third exon within *Hba-x* were annealed with each other and cloned into the *BbsI* site in PX459 V2.0. Plasmids were transformed into One-Shot Top10 Chemically Competent *E. coli* (Invitrogen) following the manufacturer’s instructions. Correct integration was confirmed via Sanger Sequencing.

A construct containing homology arms of 600 bp at the 5′ end and 1.2 kb at the 3′ end, the 2A peptide, restriction enzyme sites for fluorescent protein/localizing signal insertion (*Sal*I/*Eco*RI) and RE sites for an frt-kanamycin-frt (FKF, *Mfe*I/*Mfu*I) was ordered from GeneArt in a pMA plasmid. An FKF selection cassette was initially inserted by conventional RE cloning using *Mfe*I/*Mfu*I. The mVenus/NLS sequence was inserted by PCR cloning. The plasmid was transformed into One-Shot Top10 Chemically Competent *E.coli* (Invitrogen) and Sanger sequenced in its entirety.

Passage 14 E14Tg2a cells were plated at a density of 150,000 onto gelatin (0.1%) coated six-well plates. Plasmid HDR donor/PX459 circular DNA in a 1:1 molar ratio was added to 230 μl OptiMEM (Life Technologies) with 15 μl TransIT-LT1 (Mirus) and incubated at room temperature for 15 min, prior to being added drop-wise to cells. After 24 h culture, cells were split in a range of limiting dilutions (1:10–1:160) over six-well plates. Puromycin selection (Sigma-Aldrich) was added at 1.5 μg/ml 48 h after transfection and left for 48 h. G418 (Life Technologies) was then added at 400 μg/ml for 72 h. Media was changed at least every 48 h whilst cells were undergoing antibiotic selection. Colonies were picked into a gelatinized 96-well plate after 72 h G418 selection and grown until confluent. Following confirmation that a targeted clone was correct, it was injected into blastocysts to make chimeras. Following germline transmission of the ζ-Venus-Neo allele mice were crossed with a mouse constitutively expressing the Flp enzyme.

Fluorescence microscopy images of day 7 EBs were captured with an IX51 instrument (OLYMPUS) using a YFP filter for the and overlaid with brightfield images, obtained in parallel, using the Fiji software package^70^.

The mean fluorescence level of this tag for EBs differentiated for 4–7 days was calculated using flow cytometry with an Attune NxT instrument and proprietary software (Thermo Fisher). The mean was chosen as an appropriate metric for comparison to RNA expression levels to account for the fact that only ~40% of EB-derived cells are erythroid in nature.

For expression analyses, 2 × 10^6^ cells from whole EBs were lysed in TRI Reagent (Sigma-Aldrich) and immediately frozen at −80 °C. RNA was then extracted as detailed above. RT-qPCR was performed with Fast SYBR Green Master Mix (Thermo Fisher) and data normalized to the 18S ribosomal gene using the 2^–∆∆Ct^ method. RNA quality was routinely checked by Tapestation analysis (Agilent) and reverse transcriptase enzyme negative controls were included to confirm that DNase treatment was adequate.

Pictures of whole embryos at E10.5 were also immediately taken without any further processing on a Nikon Eclipse E600 microscope and DXM1200C camera.

Primers used for sequencing are shown in Supplementary Table 6.

### RT-qPCR analysis

Commercially validated TaqMan assay (Applied Biosystems) was used, detailed in Supplementary Table 7. *RPL13A* was used as the reference gene as previously published^71^ in humans, and *Rps18* in mice. RT-qPCR was carried out in 10 μl reaction volumes using the TaqMan Universal PCR Master Mix (Thermo Scientific), following the manufacturer’s protocol. Reactions were carried out in triplicate in 96-well plates with an ABI Prism 7500 sequence detection thermocycler (Applied Biosystems). Samples were diluted 1/10 prior to analysis with DNAse free water. Both no template and no RT (non-reversed transcribed RNA) were included in all qPCR reactions amplifying cDNA. The ΔΔCt method was used for relative quantitation.

For experiments on embryoid bodies where Venus expression was quantified, custom primers were designed and used with Fast SyBR green Mastermix (Thermo Scientific) according to the manufacturer’s instructions. Primers are shown in Supplementary Table 8.

Fetal livers from heterozygote *Hba-x*-Venus reporter mice were dissected from E12.5 embryos and cultured in Stempro (Invitrogen) supplemented with Epo (1 U/ml), SCF (50 ng/ml) and dexamethasone (1 μM). After 5 days, cell precursors were selected using anti-CD117 beads (Miltenyi Biotec) and an LS column according to the manufacturer’s instructions. Adequate purity was confirmed with flow cytometry. 50,000 cells were plated out into 96-well round bottom plates containing 200 μl of medium containing a high Epo concentration (5 U/ml) to induce differentiation and cultured for 48 h in the presence of different concentrations of HC toxin (Sigma-Aldrich-Aldrich). Plates were then analyzed on the Attune NxT instrument using an automated plate reader.

### Primary definitive erythroid cells

To isolate cells of different stages of differentiation directly from primary fetal livers at E12.5–E13.5, 5–15 fetal livers were pooled together, mechanically dissociated in staining buffer (PBS, 0.2% BSA, 5 mM glucose) and strained through a 30-μM filter. Cells were stained at 4 °C with rabbit IgG (200 μg/ml, Jackson Laboratories) to block Fc receptors. Cells were then incubated with PE-Cy7 conjugated anti-CD71 (RI7217 BioLegend, 1:2000), APC conjugated anti-Ter119 (RUO, BD Biosciences; 1:200), BB700 conjugated CD117 (104D2, BD Biosciences, 1:100), PE-conjugated Annexin V (BioLegend, 1:100), a panel of 5 FITC-conjugated lineage antibodies (anti-CD41, anti-CD45R, anti-CD3e, anti-CD11b, and anti-Ly-6G/6C, all at 1 μg/ml, all BD Biosciences), Cells were then resuspended in FACS running buffer (staining buffer with the addition of 2  mM EDTA). 0.66  μg/ml Hoechst (Invitrogen) was added prior to sorting, which was performed on a BD FACSAria Fusion machine with a 100 μm nozzle. Annexin V was only added for the experiments on the Lrf^−/−^ mice. Single color controls and FMOs were used for calibration.

### Multiplex single-cell RT-qPCR

Single erythroid cells were isolated by index-FACS from each E12.5–E13.5 embryo (12 S1 cells, 12 S2 cells, 24 S3 cells). Cells were sorted into wells of a 96-well plate containing 5.1 μl lysis buffer (5 μl 2× reaction buffer from SuperScript™ III One-Step RT-PCR with Platinum™ Taq kit (Thermo Scientific) 0.1 μl SUPERase In™ RNase Inhibitor (Thermo Scientific).

Plates were sealed, briefly centrifuged and frozen at −20 °C. TaqMan assay mastermix was made by pooling 6.6 μl of 43 TaqMan assays and adding 376 μl TE buffer. Plates were defrosted and 4.9 μl amplification mix was added to each well (1.2 μl TE buffer [Invitrogen], 2.5 μl TaqMan assay Mastermix, 1.2 μl RT/Taq enzyme mix from SuperScript™ III One-Step RT-PCR with Platinum™ Taq kit) except for one well containing S3, in which Platinum Taq was substituted for RT/Taq mix to serve as a no-RTase control. cDNA synthesis and sequence-specific preamplification were then carried out (reverse transcription at 50 °C for 15 min; RTase inactivation at 95 °C for 2 min; specific target amplification by 22 cycles of 95 °C for 15 s then 60 °C for 4 min). Taqman assays used are shown in Supplementary Table 9 and Supplementary Table 10. An RNA standard curve was prepared using the same Mastermix and amplification program.

T7 polymerase was used to in vitro transcribe RNA from cDNA clones of *Cox6c, Sec61g, Oaz1, Hba-a1, Hba-x, Hbb-bt, Hbb-y, and Hbb-bh1*. RNA was run on a 6% acrylamide TBE-urea gel (Life Technologies) and bands corresponding to the full-length transcript were isolated from the gel. RNA was extracted by incubating crushed gel pieces with elution buffer (500 mM ammonium acetate, 1 mM EDTA–KOH pH 8.0) for 3 h at room temperature before ethanol precipitation, resuspension in TE buffer, and quantification using the RNA Quantifluor assay (Promega) and RNA Tapestation (Agilent). Each RNA was diluted to 16.8 pM concentration and the eight transcripts were pooled, aliquoted, and stored at −80 °C.

When performing reverse transcription and pre-amplification on sorted single cells, one aliquot of RNA standard was also defrosted and diluted 10×. Five more serial dilutions were created by diluting 6x in TE buffer, then 2 μl was taken from each of the six standard curve dilutions and pre-amplified using the same mastermix as used for single cells Plates were stored at −20 °C until analysis. Pre-amplified cDNA was diluted 5× with TE buffer and analyzed using Universal PCR Master Mix (Applied Biosystems) and individual TaqMan gene expression assays (Life Technologies), on the Biomark System (Fluidigm) using two 192.24 Dynamic Arrays as per the manufacturer’s protocol, except that elongation time was increased from 60 to 75 s to accommodate longer custom gene-expression assays for *Hba-a1/2* and *Hbb-bt/s/1/2*.

Analysis was performed using Python (v3.5.2; Matplotlib v2.0.2; Numpy v1.14.5; Pandas v0.23.3; scikit-learn v0.19.2; Scipy v1.1.0). Ct values beyond the limit of detection or marked as “Fail” by the instrument were set to the limit of detection value (Ct = 40). Raw Ct values were transformed into expression space by calculating 2^−Ct^. For the Lrf^−/−^ knockout experiments expression of all genes was scaled to a range of 0–1, then the mean of all scaled expression values was calculated for each cell and used to normalize expression values. For the experiments on the *Bcl11a*^*Δ10kb/Δ10kb*^mice, then three housekeeping genes (*Cox6c*, *Myl6*, and *Sec61g*) were run on both 192.24 chips. The expression of these three genes was scaled to a range of 0–1, then the mean of the three scaled expression values was calculated for each cell and used to normalize expression values for the remaining 21 assays on each chip.

### Transgenic enhancer assays

Enhancer activity was assayed using an established mouse transgenic system^26,27^ using a vector containing a candidate enhancer, a minimal promoter, and the LacZ gene stably integrated into the mouse genome via pronuclear injection. Coordinates of enhancer elements given in Supplementary Table 2. To visualize LacZ staining on histological sections, embryos were fixed in 4% paraformaldehyde at 4 °C, washed three times in phosphate-buffered saline, embedded in OCT-compound (Sakura Finetek), and cut on a cryostat (Leica, Deerfiled, IL) at 10 µM. Specimens were viewed and photographed using a Leica MZFLIII microscope and DFC300F camera. Images were viewed and stored using Openlab software (Improvision).

### DNAseI assay

The DNaseI assay was performed as previously published^20^. Library preparation was performed using the NEBNext DNA library preparation (E6000S) with adaptations for low input DNA and size selection. Adapter stocks were diluted 1:10 and primer stocks were diluted 1:10. For size selection, libraries were run on a 2% agarose gel (Biorad), stained with Ethidium Bromide (Sigma) and the required size (145–450bp) excised. DNA was extracted using a gel extraction kit (Zymo).

### DNaseI digital footprinting

DNase digital footprints were called using the standard parameters of pyDNase (version 0.1.7) Python package which implements the Wellington algorithm^72,73^.

### Chromatin immunoprecipitation analysis followed by high throughput sequencing (ChIP-seq)

Chromatin immunoprecipitation (ChIP) was performed on 3–5 × 10^6^ cells per replicate using the ChIP Assay Kit (Cat. No. 17-295, Millipore). All stages beyond the fixation stage used kit solutions as per the manufacturer’s instructions. A single 10 min 1% formaldehyde fixation was used for all antibodies. The following antibodies and amounts/dilutions were used: CTCF 1:100 (Cat. No. 07-729, Millipore), H3K27ac 1:1000 (Cat. No. ab4729, AbCam), H3K4me1 1:5000 (Cat. No. 07-436, Merck), H3K4me3 1:200 (Cat. No. ab8580, AbCam), GATA1 1:200 (Cat. No. ab11852, AbCam), H2AK119ub 1:100 (Cat No. #8240, CST) H3K27me3 1:100 (Cat. No. 9733, Cell Signaling Technologies). The cross-linking reaction was quenched by the addition of glycine (Sigma-Aldrich) to a final concentration of 130 mM), then washed once in PBS and snap-frozen. Samples were stored at −80 °C prior to further processing. Chromatin fragmentation was performed using the Covaris S220 Focused ultra-sonicator with the following settings (Duty Cycle: 2%, Intensity: 3.0, Cycles/burst: 200, Power mode: Freq. Sweeping, Duration: 120 s, Temp: 6 °C, Batches: 4) to obtain an average fragment size between 200 bp and 500 bp. DNA libraries for sequencing were prepared with the NEBNext Ultra II DNA library prep kit (New England Biolabs) and sequenced on the Illumina platform.

### Bisulfite sequencing

High-quality genomic DNA was bisulfite-converted using the EZ DNA Methylation Gold Kit (Zymo Research) following the manufacturer’s instructions. Enzymatically methylated human genomic DNA was used as the methylated control (Millipore). Nested primer sets were designed to cover the *HBZ* gene and its promoter (Supplementary Table 11). PCR was performed with the HotStart Taq Polymerase according to the manufacturer’s instructions. Products were then run out on a 1% Agarose gel, and gel extracted using the Zymoclean Gel DNA recovery kit (Zymo Research) according to the manufacturer’s instructions. Libraries were generated using a NEBNext Ultra DNA Library Prep Kit (NEB) according to the manufacturer’s instructions and sequenced using an Illumina Miseq v2 500 cycle kit.

### Analysis of the chromatin landscape using ATAC-seq

ATAC-seq was performed as published^74^. 10,000–50,000 cells were used per biological replicate where possible; if cells numbered <10,000 an optimized low cell number protocol was used. The low cell number protocol uses proportionally less lysis buffer and Tn5 transposase^75^. After spinning at 500 × *g* for 10 min and removing the supernatant, cells were permeabilised and tagmented with Dig-transposition buffer (25 μl 2 × TD buffer [Illumina], 2.5 μl Tn5 Transposase, 0.5 μl 1% digitonin and 22 μl H_2_O) for 30 min. After the transposition step, samples were processed as per larger cell numbers.

### Analysis of ChIP-seq and ATAC-seq data

Sequencing quality was assessed by FASTQC. ChIP-seq and ATAC-seq data were aligned to the mm9 (for mouse) and hg19 (for human) data using a customized in-house pipeline^76^ (script: https://github.com/Hughes-Genome-Group/NGseqBasic/releases). Output BAM files were sorted and indexed using Samtools, then normalized using Deeptools to RPKM (–normalizeUsingRPKM), then converted to a bigwig using  UCSC tools. Data were visualized in the UCSC genome browser. Published datasets were downloaded from the NCBI Gene Expression Omnibus (GEO) database. FASTQ files were then processed as described.

### Next generation Capture-C

Next generation Capture-C was performed as previously described^12,19,30^. Material was sequenced using the Illumina MiSeq platform with 150-bp paired-end reads (300-bp V2 chemistry). Data were analyzed using analysis scripts (github.com/Hughes-Genome-Group/CCseqBasicF), and custom perl and R scripts were used to normalize data and generate differential tracks (github.com/djdownes/CaptureCompare)^76^.

### Super-enhancer analysis

Enhancer and promoter annotations were generated using GenoSTAN^24^. All ATAC-seq hypersensitive regions were sub-classified based upon their H3K4me1, H3K4me3, H3K27ac, promoter annotation, and CTCF binding.

Super-enhancers were called using the ROSE tool as described^25^. Individual enhancers were stitched together to form a single enhancer domain. Stitched enhancers were then ranked for H3K27ac signal, which was normalized to a maximum value of 1.0. Regions ± 250 bp of annotated TSS were excluded. Enhancer domains were called super-enhancers if H3K27ac signal was above a threshold placed where the tangent to the graph ranking enhancers was equal to 1.

### Micro Capture-C

This was performed as described^31^ were fixed for 10 min at room temperature with formaldehyde (2% (vol/vol) and quenched with glycine (130 mM). The cell pellet was washed and reconstituted in 1 ml PBS and digitonin 0.005% (wt/vol) (Sigma D-141). Permeabilised cells were centrifuged (300 × *g*, 5 min) resuspended in micrococcal nuclease buffer (Tris–HCl pH 7.5 10 mM; CaCl_2_ 1 mM). Cells were digested with MNase (NEB M0247) typically ranging from 5 to 20 Kunitz U for a reaction volume of 800 µl containing 2–3 × 10^6^ cells 1 h at 37 °C on an Eppendorf Thermomixer at 800 rpm. The reaction was quenched with ethylene glycol-bis(2-aminoethylether)-N,N,N′,N′-tetraacetic acid (EGTA) (Sigma E3889) to a final concentration of 5 mM. 200 µl was removed as a control to measure the digestion efficiency. The reaction was centrifuged (5 min, 300 × *g*) and the digestion buffer was discarded. Cells were resuspended in DNA ligase buffer (Thermo Scientific; final concentrations Tris–HCl pH 7.5 40 mM, MgCl_2_ 10 mM, DTT 10 mM, 5 mM ATP) supplemented with dNTPs (final concentration 400 µM of each of dATP, dCTP, dGTP and dTTP (Thermo Fischer R0191)) and EGTA 5 mM. T4 Polynucleotide kinase PNK (NEB M0201L) and DNA Polymerase I Large (Klenow) Fragment (NEB M0210L) were added to final concentrations of 200, 100 U/ml, respectively, and the reaction was incubated at 37 °C for 1 h. T4 DNA ligase (Thermo Scientific EL0013) was added to a final concentration of 300 U/ml and the reaction was incubated at 16 °C overnight using an Eppendorf Thermomixer at 800 rpm. The chromatin was decrosslinked with proteinase K at 65 °C (>2 h) and the DNA was extracted. Oligonucleotide capture was performed as previously described (Davies et al., Nature Methods 2016). Data were analyzed with a custom analysis pipeline specifically developed for MCC data analysis^31^.

*URLs*. UCSC Genome Browser Track Hub containing normalized replicate ATAC-seq and normalized NG CaptureC data without windowing: http://sara.molbiol.ox.ac/public/hugheslab/Primitive_alpha_globin/hub.txt. Previously published data used are shown in Supplementary Table 12.

### Reporting summary

Further information on research design is available in the Nature Research Reporting Summary linked to this article.

## Supplementary information

Supplementary Information

Reporting Summary

## Data Availability

Source data availability: Raw sequence data generated are available on the Gene Expression Omnibus (GEO) database under the following accessions: GSE108434 (Open chromatin, Capture-C and epigenetic data in wild type primitive murine cells), GSE174110 (Open chromatin data in mouse models), GSE153256 (Micro Capture-C in definitive murine erythroid cells), and GSE173419 (Open chromatin and epigenetic data in edited HUDEP2 cells). These data are also available in the following UCSC datahub: https://datashare.molbiol.ox.ac.uk/public/project/fgenomics/publications/King_2021_Zeta_Activation/hub.txt. Source Data for all figures are provided in the Source Data file. All other data supporting the findings of this study are available from the corresponding author on request. Published datasets analyzed (see Supplementary Table 12 for details): GSE137477, GSE27921, GSE97871, GSE 74977, GSE103445, GSE 104676, GSE36944, GSE 71422. Source data are provided with this paper.

## Source data

Source Data

## Code availability

ChIP-seq and ATAC-seq data were aligned to the mm9 (for mouse) and hg19 (for human) data using a customized in house pipeline^76,77^ (script: https://github.com/Hughes-Genome-Group/NGseqBasic/releases). Next generation Capture-C data were analyzed using analysis scripts (github.com/Hughes-Genome-Group/CCseqBasicF), and custom perl and R scripts were used to normalize data and generate differential tracks (github.com/djdownes/CaptureCompare)^76^.
