# Supplementary material for: Reactivation of a developmentally silenced embryonic globin gene
Source: Nat Commun. 2021 Jul 21;12:4439. doi: 10.1038/s41467-021-24402-3 (PMC8295333; doi:10.1038/s41467-021-24402-3)
Supplement: Supplementary file 1 — Supplementary Information [file 41467_2021_24402_MOESM1_ESM.pdf]

Supplementary Figures and Tables

Supplementary Figure 1

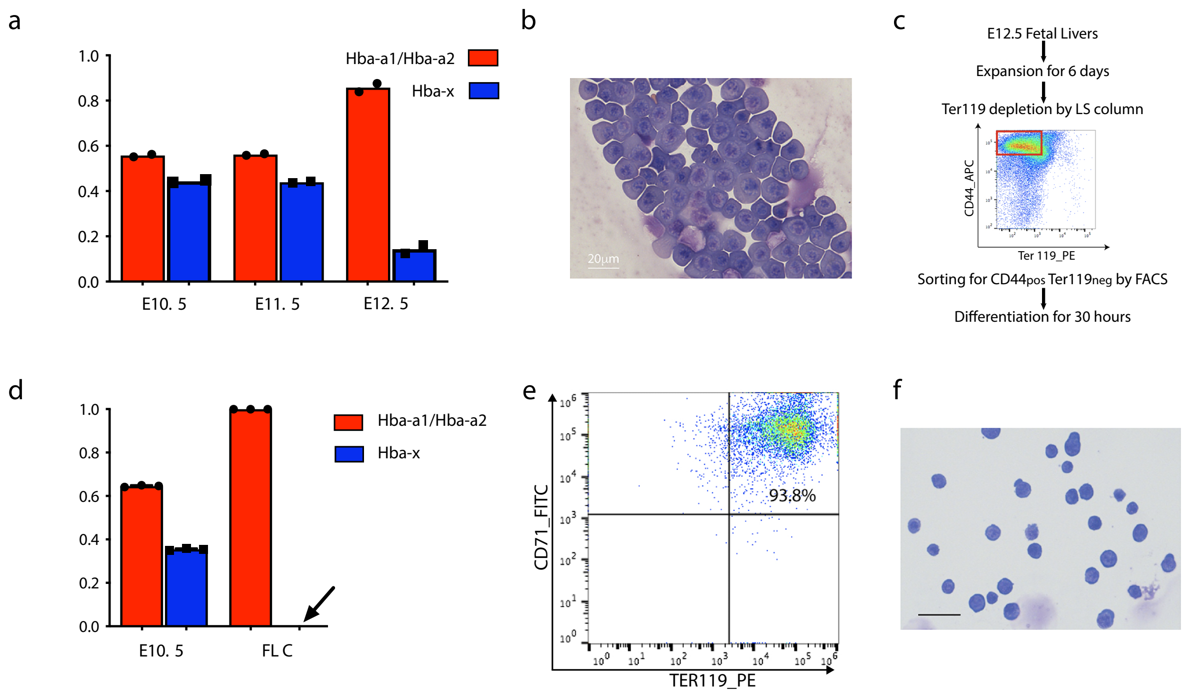

Isolation of primitive and definitive erythroblasts. a. Gene expression levels of the α-like globins (*Hba-x* and *Hba-a1*/*Hba-a2*) from mouse primary primitive erythroid cells measured using real-time qPCR and expressed as a ratio of the total α-like globin. n=2 biologically independent samples. b. Representative cytospin stained using a modified Wright's stain of E10.5 erythroid cells demonstrating a homogenous population of intermediate erythroblasts (scale line indicates 20μm, experiment repeated 9 times). c. Strategy for differentiating erythroid cells. Details of expansion and differentiation media are included in materials and methods. d. qRT-PCR data confirms absence of *Hba-x* expression. Bars represent the mean and the error bars represent the standard deviation (s.d.) of technical replicates (n=3). e. Flow cytometry at 30 hours shows a synchronous population of erythroid cells which are CD71 and Ter119 positive; morphologically these are intermediate erythroblasts. f. Representative cytospin of cells obtained in e, stained using a modified Wright's procedure (scale line indicates 20μm, experiment repeated 9 times). Source data are provided as a Source Data file.

Supplementary Figure 2

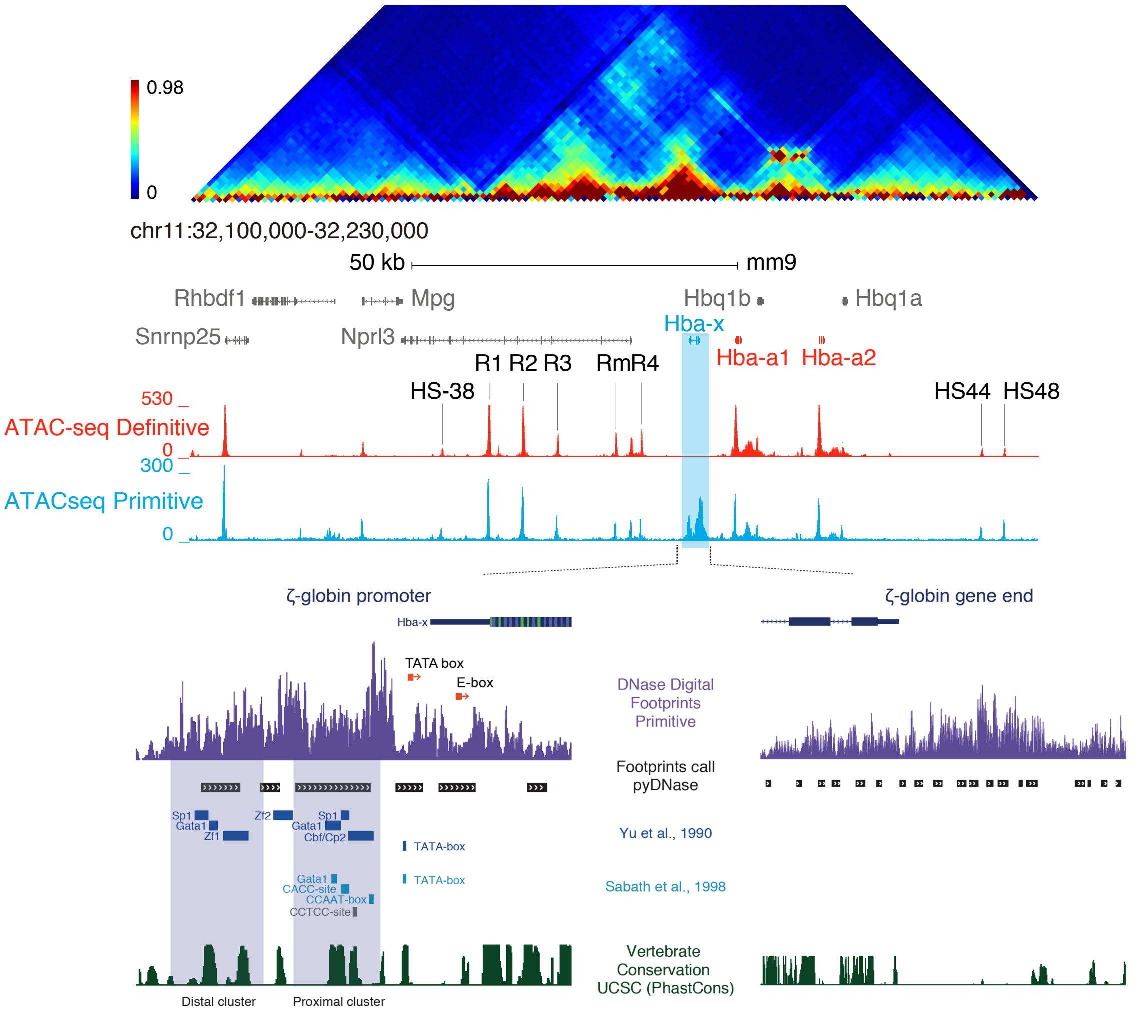

DNase I footprinting of the ζ-globin gene promoter. Upper panel, Tiled-C interaction map showing interactions across the α-globin locus in definitive erythroblasts (from ref 16). Middle. DNase I hypersensitivity with footprinting was performed to generate a list of potential activating transcription factors of ζ-globin in mice from both the promoter and the 3′ UTR region (Supplementary Table 1). Lower, footprints were called using pyDnase. Vertebrate sequence conservation is shown via PhastCons[1].

Supplementary Figure 3

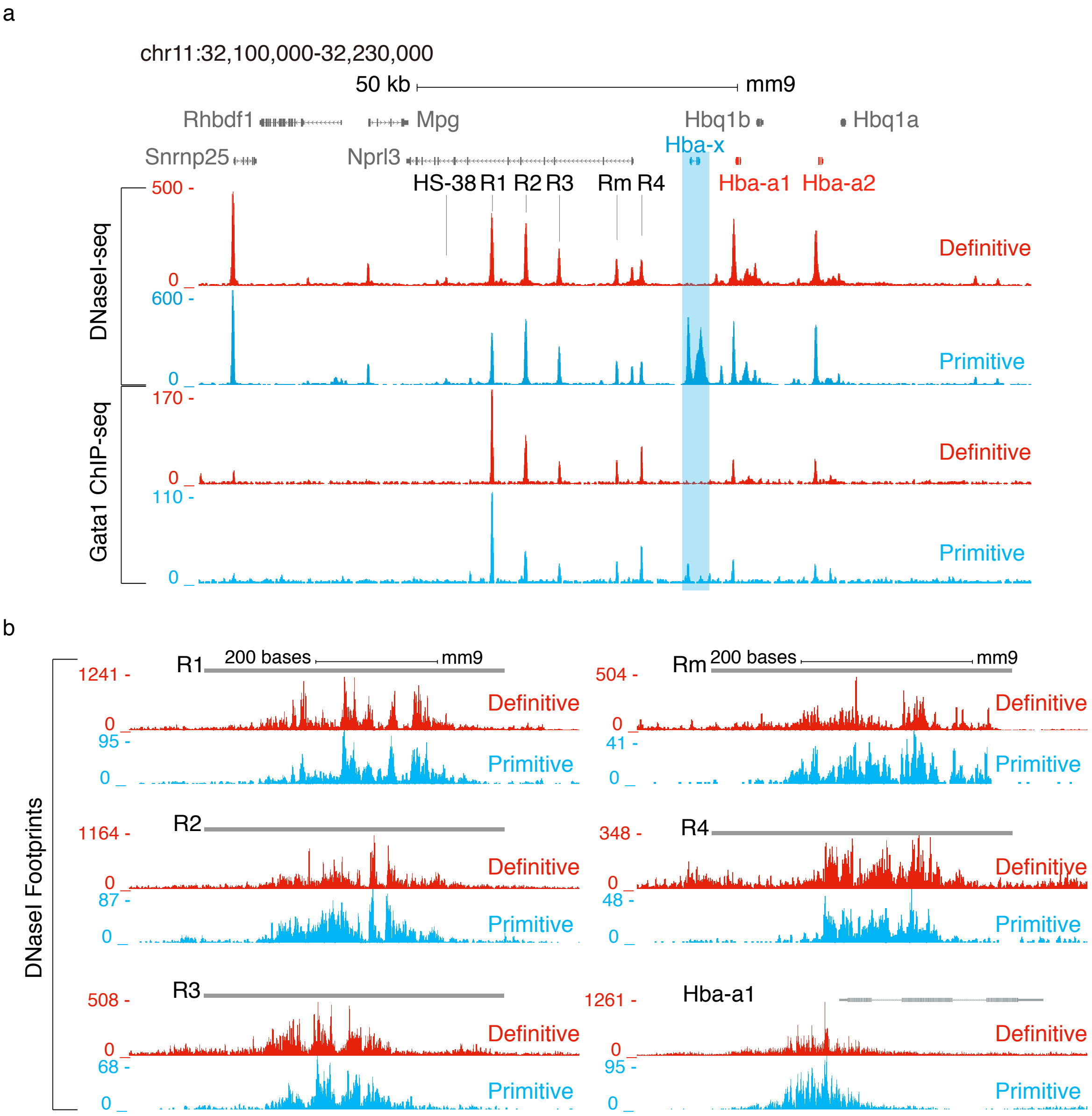

Gata1 binding and DNAseI Footprinting in primitive and definitive erythroid cells. a. ChIPseq of Gata1 shows binding at the ζ-globin gene promoter is lost in definitive erythropoiesis. b. DNaseI footprinting shows a similar pattern at the α-globin enhancer elements in both primitive and definitive erythroid cells with minor changes present at the Rm element, implying similar transcription factor binding.

Supplementary Figure 4

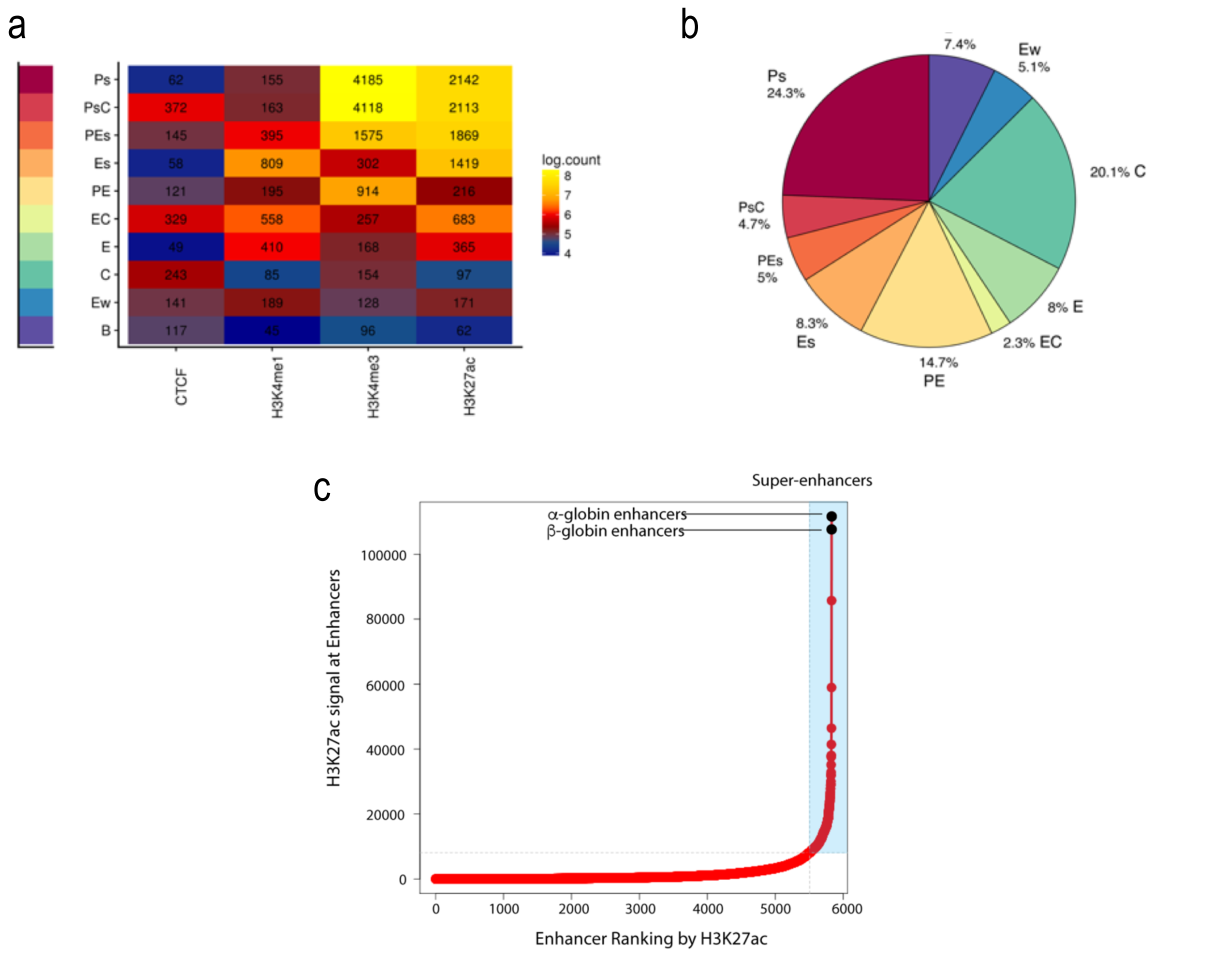

The α-globin enhancer is a super-enhancer in primitive erythroblasts. a. 10 Chromatin states were identified using the read count for CTCF, H3K4me1, H3K4me3 and H3K27ac: Ps (strong promoters), PsC (strong promoters with CTCF binding), PEs (promoters with an enhancer mark), Es (strong enhancers), PE (promoters with an enhancer mark), EC (enhancers with a CTCF mark), E (enhancers), C (CTCF sites), Ew (weak enhancers) and B (background). b. The proportion of elements associated with each chromatin state identified. c. 327 super-enhancers were identified in primitive erythroid cells. The two top-ranking super-enhancers are the α-globin and β-globin enhancer clusters.

Supplementary Figure 5

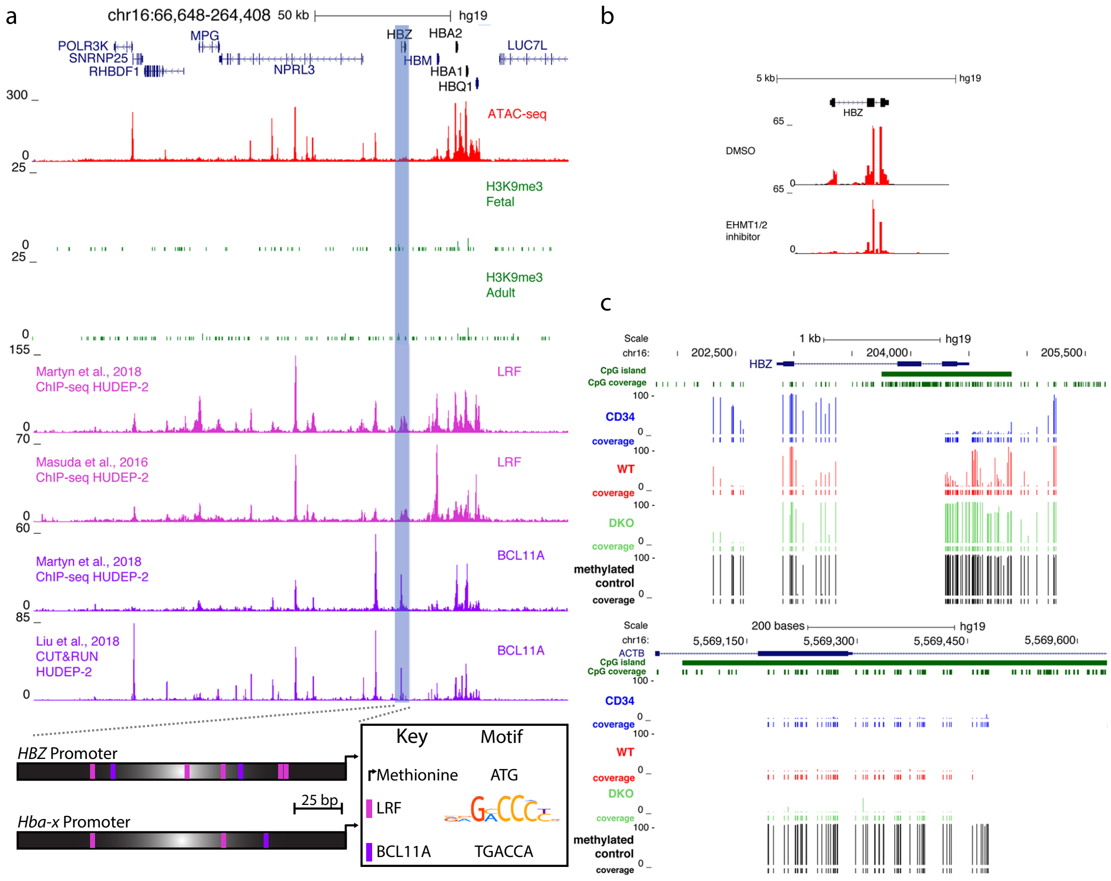

The ζ-globin gene is not silenced by H3K9me3 or DNA methylation, however it is bound by the repressors LRF and BCL11A. a. Upper, ChIP-seq for H3K9me3 across the α-globin locus in primary erythroid cells derived from fetal and adult CD34$^+$ cells[2]. ChIP-seq and CUT&RUN data for LRF and BCL11A binding across the α-globin locus; the relevant publication, cell type and technique used are shown. An ATAC-seq track for HUDEP-2 WT cells is also shown for orientation. Lower, conservation of binding sequences for the repressors of zeta-globin gene expression LRF1, and BCL11A in the human and mouse ζ-globin promoters. The motifs used for LRF are from HOCOMOCO (https://hocomoco11.autosome.ru/) and the BCL11A motif is from references 3 and 4. c. Normalised RNA-seq generated following addition of DMSO control (top track) and the EHMT1/2 (G9a) inhibitor UNC0638 (bottom track)[5]. There is no increase in ζ-globin expression following EHMT1/2 inhibition indicating that H3K9me2 does not play a role in repression. c. Bisulfite sequencing across the *HBZ* gene in erythroid cells derived from primary CD34 cells, WT HUDEP-2 cells and the DKO HUDEP-2 clone in which lack LRF and BCL11A function. The promoter of the active *ACTB* gene serves as an unmethylated control. In primary erythroid cells derived from adult CD34+ cells, four CpGs are methylated and two are unmethylated,

whereas in HUDEP-2 cells (which still express trivial amounts of ζ-globin), all but one is unmethylated. As to why these are different is unclear and may relate to aberrant methylation due to HUDEP-2 immortalisation, or that HUDEP-2 cells are derived from fetal CD34+ cells rather than adult. However, given ζ-globin is still effectively silenced in WT HUDEP-2 cells, DNA methylation at the promoter does not appear to play a silencing role.

Supplementary Figure 6

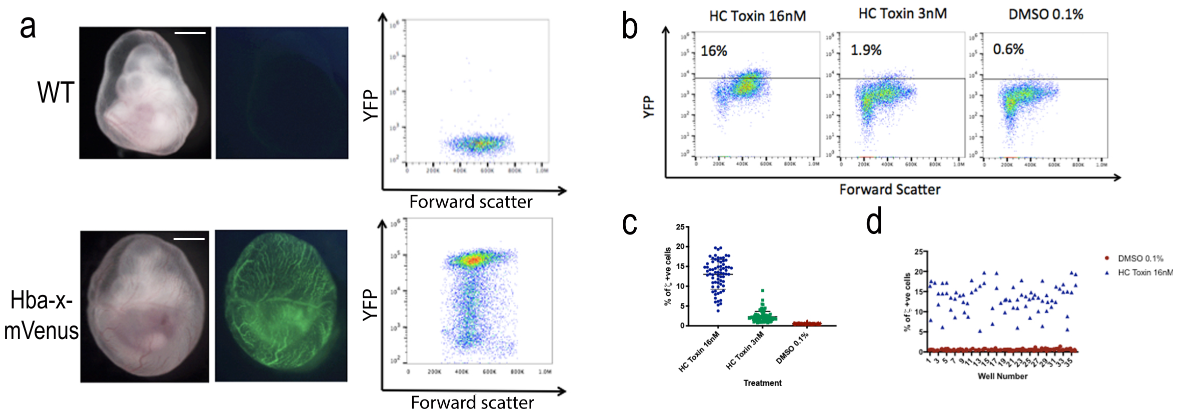

HDAC inhibition by HC-Toxin reactivates ζ-globin transcription. a. Microscopy of Hba-x-mVenus/WT and WT/WT littermate embryos under incident light (left panels) and ultra violet illumination with a YFP filter (middle panels), right panels show representative flow cytomety plots for erythroid cells derived from E10.5 embryos. A clear fluorescent signal is seen in th e ζ-Venus heterozygous embryos. scale bars indicate 1mm. Repeated independently 3 times. b. Representative flow cytomety plots for erythroid cells derived from fetal liver cultured from E12.5 embryos, which do not normally express ζ-globin, treated with 16nm or 3nM of HC-Toxin, which induces ζ-globin expression (evidenced by mVenus) in a dose dependent manner. c. There is a significant increase in fluorescence in cells treated with HC-Toxin treatment across one hundred replicates (P <0.0001 for 3nM and 16nM), bars show median expression and SD. d. Scatter plot showing the percentage of mVenus positive definitive erythroblasts when treated with 16nM of HC toxin compared to the negative control (DMSO 0.1%). Source data are provided as a Source Data file.

Supplementary Figure 7

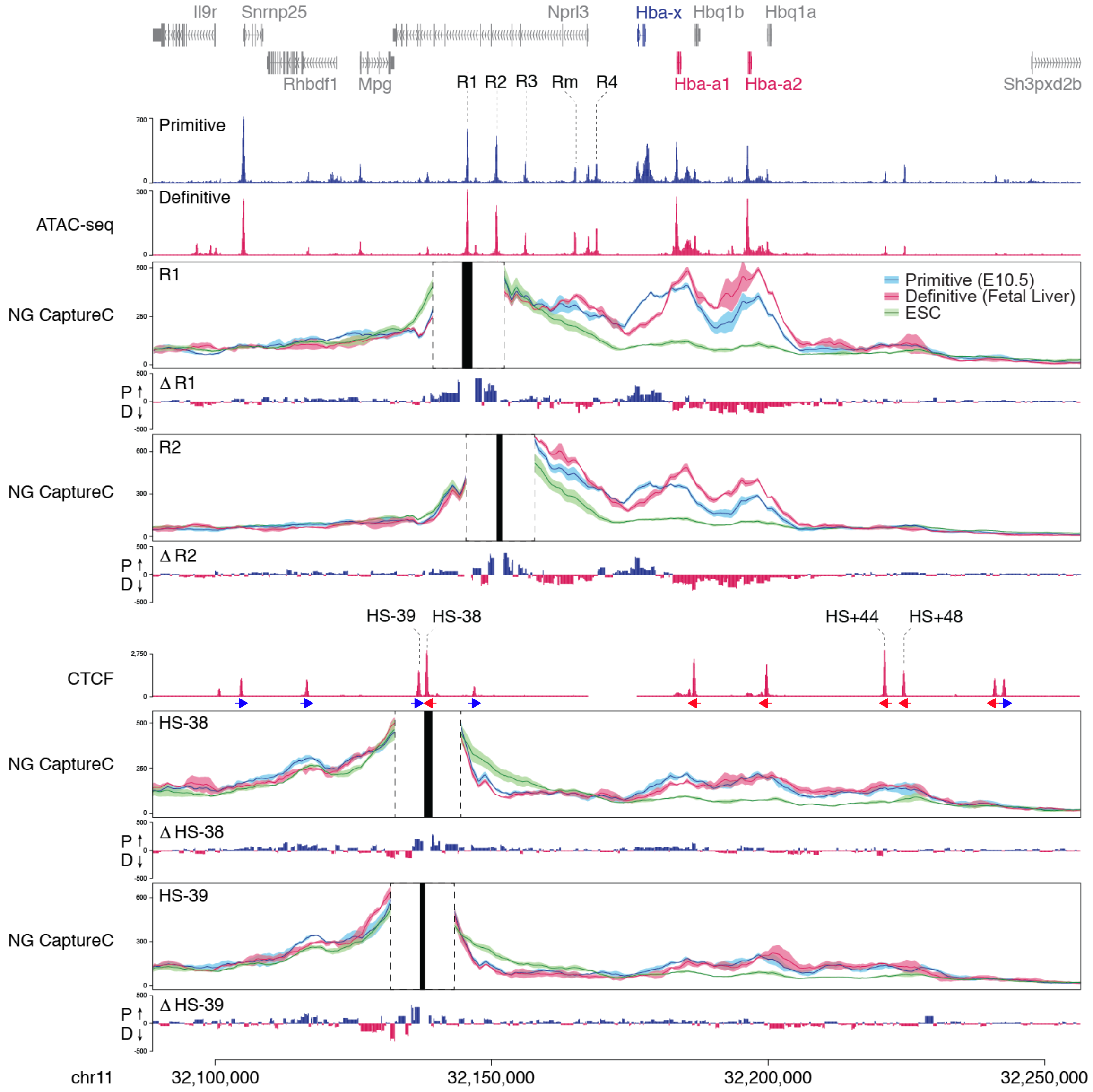

The α-globin self-interacting domain is highly similar in primitive and definitive erythroblasts. Overlaid normalized Capture-C data from the viewpoints of R1 and R2 and the HS-38 and HS-39 CTCF sites. The mean, plus and minus one standard deviation (s.d.), of sliding 5 kb windows are visualized. Differential tracks (ΔCapture-C) show a subtraction (Primitive - Definitive) of the mean number of meaningful interactions per restriction fragment. Grey vertical bars indicate the position of the viewpoint; the enhancers and boundary CTCF elements are shown above an ATAC-seq track from primitive erythroid cells. A ChIP-seq track demonstrating CTCF boundary sites from definitive erythroid cells is shown along with the orientiation of CTCF sites[6]. All tracks are merged data from three independent experiments.

117 **Supplementary Figure 8**

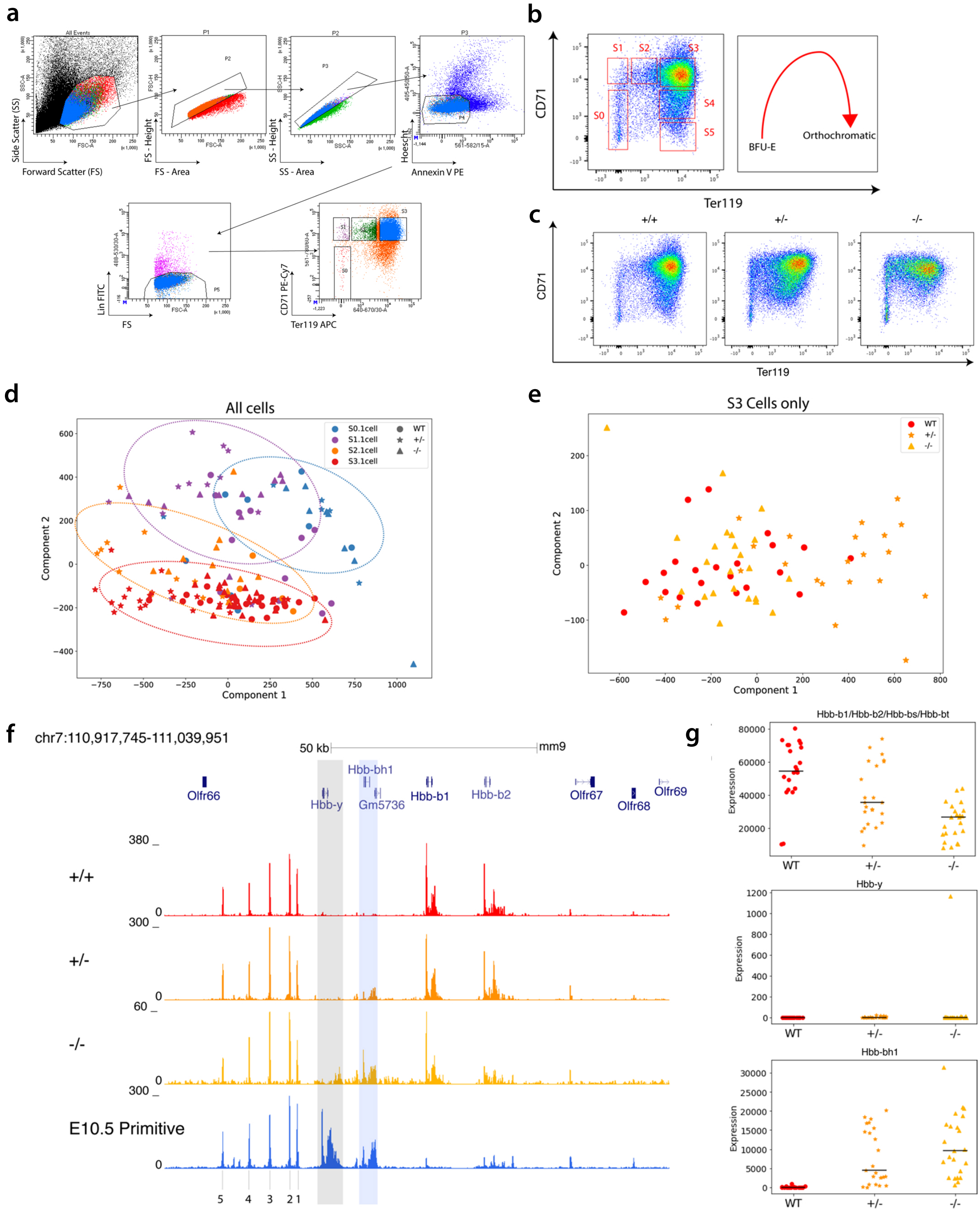

118

Embryonic and fetal β-like globin expression does not result from a differentiation block in Lrf mutant erythroblasts. a. Erythroid differentiation in freshly isolated fetal liver (E12.5), defined by the CD71/Ter119 flow cytometric profile. Cells can be separated into six sub-sets (S0-S5), corresponding to increasing erythroid differentiation. b. Representative plots of erythroid differentiation in *Lrf*$^{Flox/Flox}$(+/+), *Lrf*$^{Flox/-}$ (+/-) and *Lrf*$^{-/-}$(-/-) from ~E12.5 fetal livers. There is a differentiation block in the *Lrf*$^{Flox/-}$ and the *Lrf*$^{-/-}$ erythroid cells. c. PCA analysis excluding the globin genes demonstrates that S0, S1, S2 and S3 cluster together. d. When considering one population (the S3 population) the WT, heterozygote and homozygote *Lrf* $^{-/-}$ cells do not cluster together, confirming the differentiation stage is similar across these cell populations. e,f. Chromatin accessibility and single cell expression at the β-globin locus in *Lrf*$^{Flox/Flox}$ (+/+), *Lrf*$^{Flox/-}$ (+/-) and *Lrf*$^{-/-}$ (-/-) primary murine erythroid cells from the S3 population. Only *Hbb-bh1* is derepressed. The *Hbb-y* gene and *Hbb-bh1* genes are highlighted in grey and blue respectively.

133 **Supplementary Figure 9**

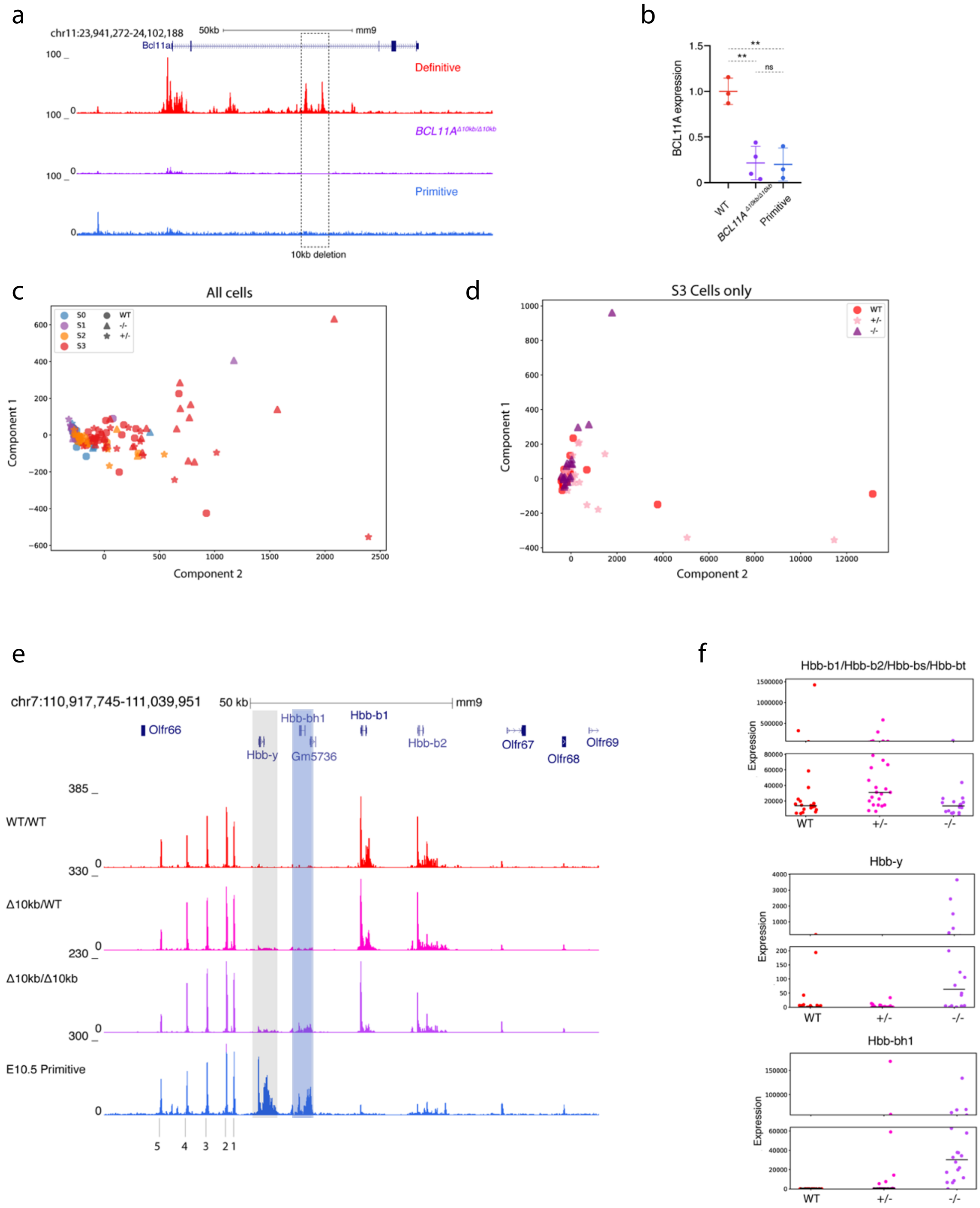

134

Characteristion of reduced *Bcl11a* expression and cell identity in *Bcl11a*$^{\Delta10kb/\Delta10kb}$ definitive erythroblasts. a,b. Chromatin accessibility and *Bcl11a* expression in WT definitive, primitive (E10.5) and *Bcl11a*$^{\Delta10kb/\Delta10kb}$ definitive erythroid cells. *Bcl11a* expression was performed in bulk populations on steady-state RNA, normalized to R18s, and then normalized to WT. Experiments were performed in ≥3 independent biological replicates (WT, N=3; BCL11A Δ10kb/10kb, N=4; E10.5, N=3). Statistical analysis is by one-way ANOVA with a Bonferroni correction for multiple comparisons; the individual data points and SEM are shown. ** denotes $p<0.01$. Bars show mean +/- SEM. Definitive erythroid cells used for experiments were Ter119 selected erythroid cells from adult spleens. c. PCA analysis excluding the globin genes demonstrates that there is no clustering based on genotype; this is also true when considering the S3 population only (d). This confirms the cells analysed are definitive erythroid. e, f. Chromatin accessibility and single cell expression at the β-globin locus in *Bcl11a*$^{WT/WT}$ (WT/WT), *Bcl11a*$^{\Delta10kb/WT}$ (Δ10kb/WT) and *Bcl11a*$^{\Delta10kb/\Delta10kb}$ (Δ10kb/Δ10kb) erythroid cells from the S3 population. Both the embryonic genes are de-repressed. The *Hbb-y* gene and *Hbb-bh1* genes are highlighted in grey and blue respectively. Source data are provided as a Source Data file.

Supplementary Figure 10

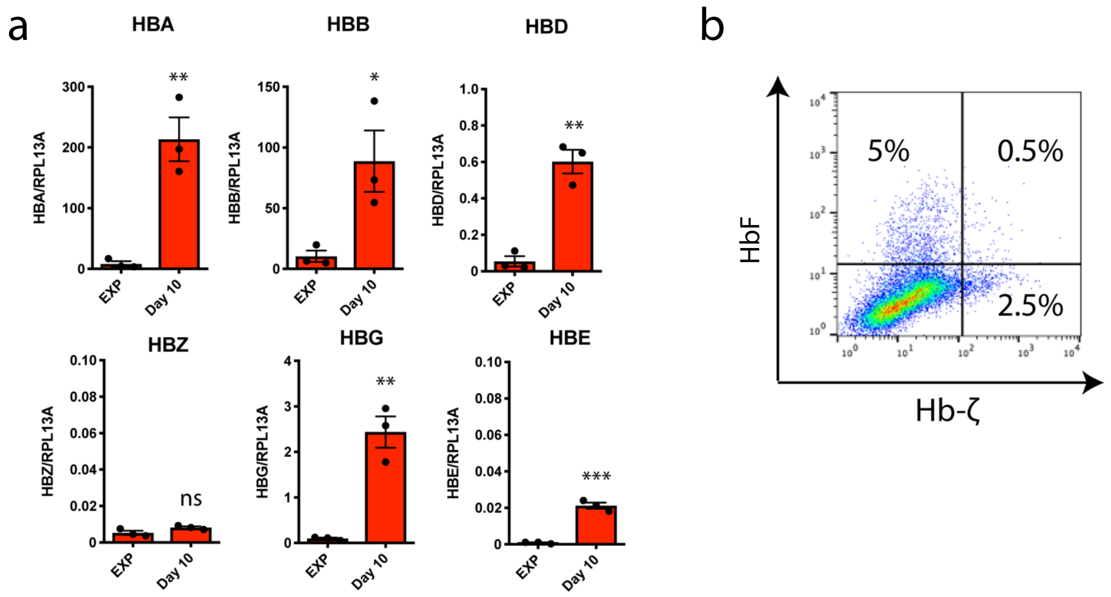

Characterisation of globin expression in WT HUDEP-2 cells. a. Expression analysis for *HBA*, *HBB*, *HBD*, *HBZ*, *HBG* and *HBE* from HUDEP-2 cells in expansion phase and at Day 10 of differentiation, normalized to *RPL13A*. * denotes $p<0.05$, ** denotes $p<0.01$; *** denotes $p<0.001$. Significance was determined by a two-tailed Student's t-test. N=3 independent experiments (p values: HBA, p 0.0048; HBB, p 0.038; HBG2, 0.0025; HBD, 0.0015; HBE, 0.0003). Error bars show the mean +/- SEM. b. Representative flow cytometry on HUDEP-2 cells for HbF and ζ-globin. There is minimal fetal and embryonic globin expression or protein production in HUDEP-2 cells. Source data are provided as a Source Data file.

Supplementary Figure 11

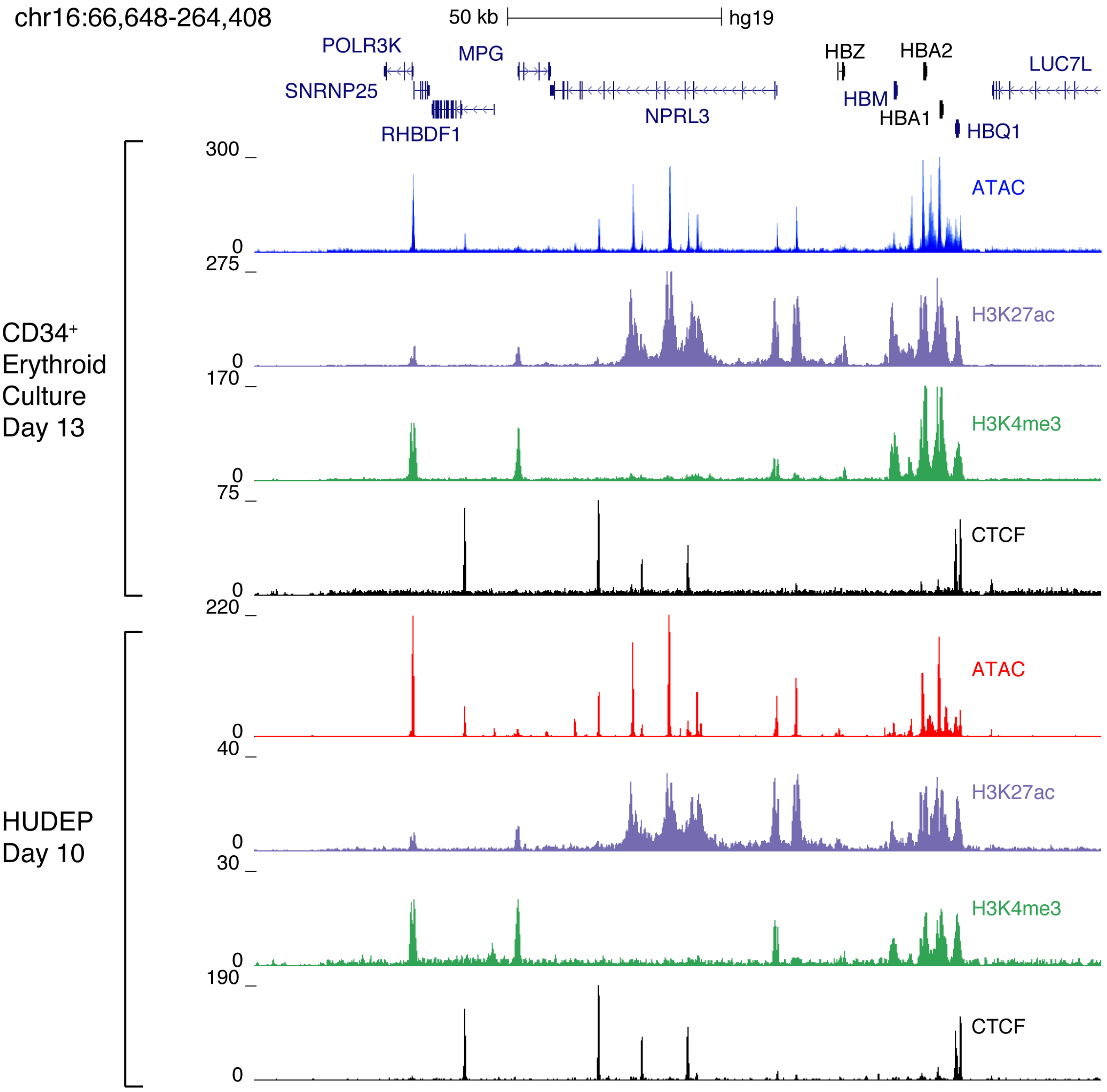

HUDEP-2 cells accurately model the α-globin locus. Histone marks and CTCF binding for primary erythroid cells and HUDEP-2 cells at the α-globin locus. Primary erythroid cell data are from a $CD34^+$ three-phase erythroid culture system. HUDEP-2 ChIP-seq data consist of a single replicate performed on Day 10 of the HUDEP-2 culture system; ATAC-seq data are in triplicate. Data are normalized by RPKM.

Supplementary Figure 12

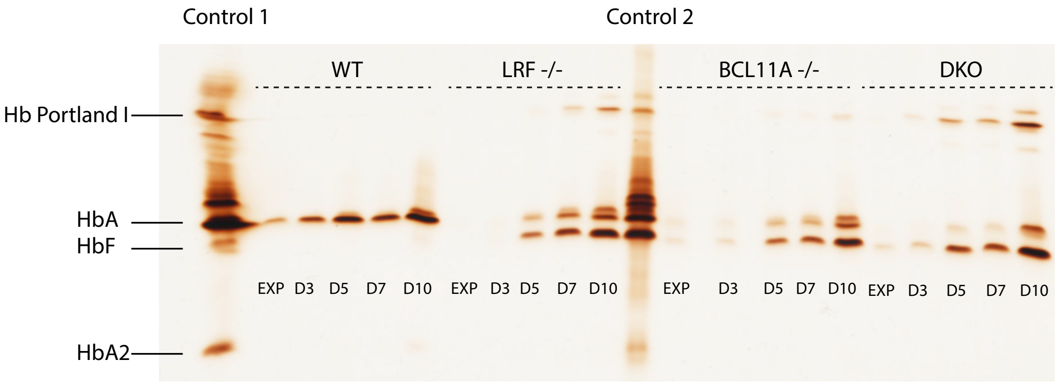

Isoelectic focusing of extracts from WT, LRF$^{-/-}$, BCL11A$^{-/-}$ and DKO HUDEP2 cells through differentiation shows detectable Hb Portland I ($\zeta_2\gamma_2$) in the mutant cells. DKO cells have the highest amount of Hb Portland I. Exp denotes expansion media; D3, D5, D7 and D10 denotes the day of differentiation. Two controls are shown. Control 1 is a transfused infant with ζ --^{SEA}/ζ --^{SEA} some of these patients have ζ-globin expression at birth[7]. Control 2 is a compound heterozygote for mutations in the *KLF1* transcription factor which has been extensively characterised and is known to express ζ-globin[8]. Source data are provided as a Source Data file. Repeated independently 3 times.

Supplementary Figure 13

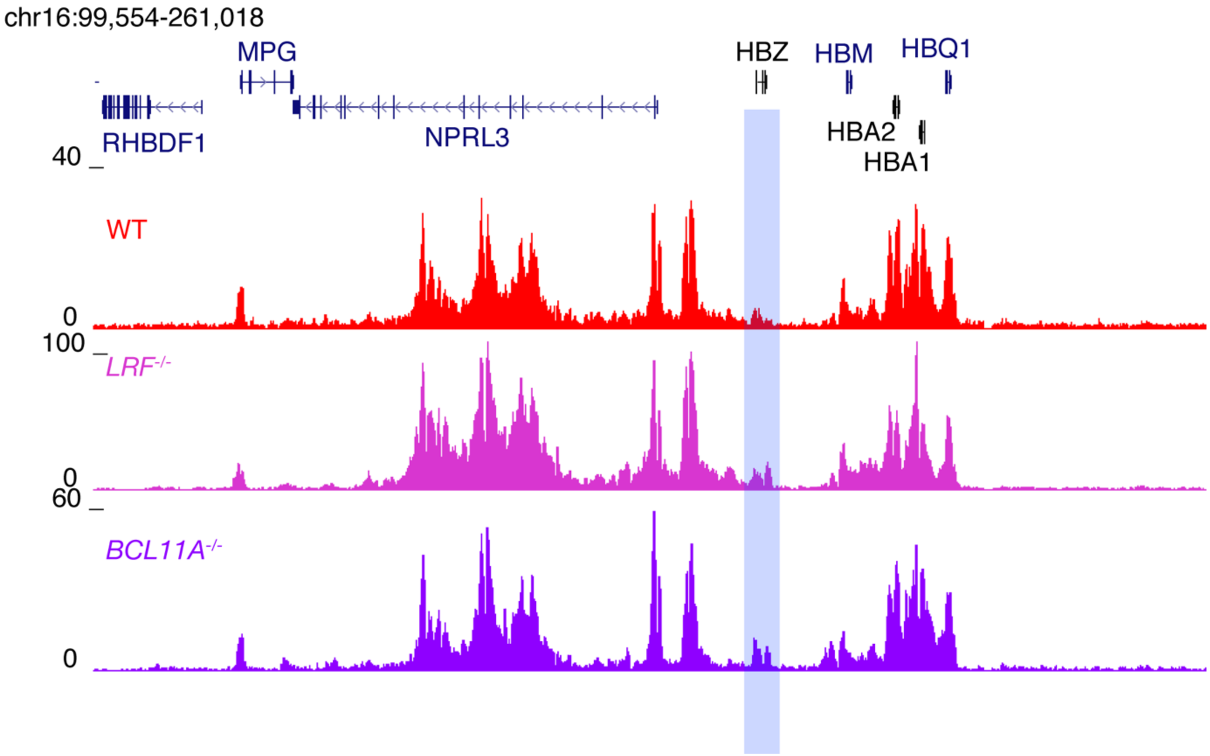

Knockout of *LRF* and *BCL11A* individually leads to a small increase in histone acetylation at the ζ-globin gene. ChIP-seq tracks of H3K27ac for WT, *LRF*$^{-/-}$ and *BCL11A*$^{-/-}$ HUDEP-2 cells (single replicate). The ζ-globin gene is highlighted in blue.

Supplementary Figure 14

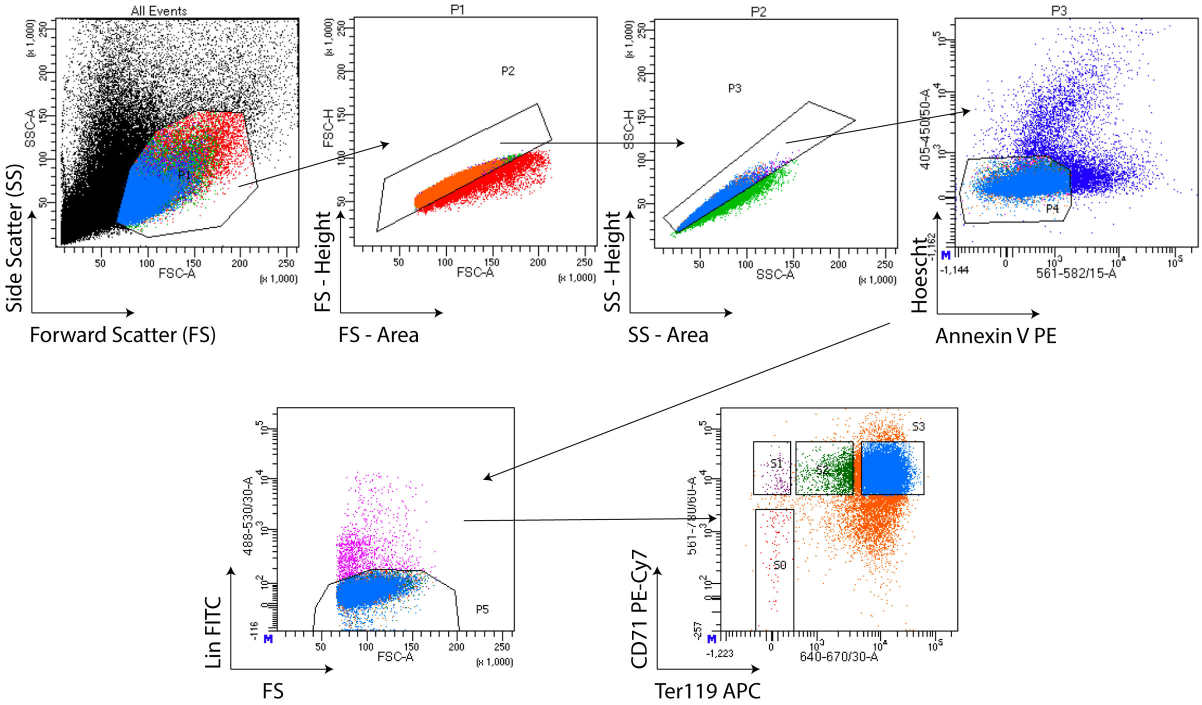

Sorting strategy for experiments presented in Figure 6 and Supplementary Figures 8 and 9

Supplementary Figure 15

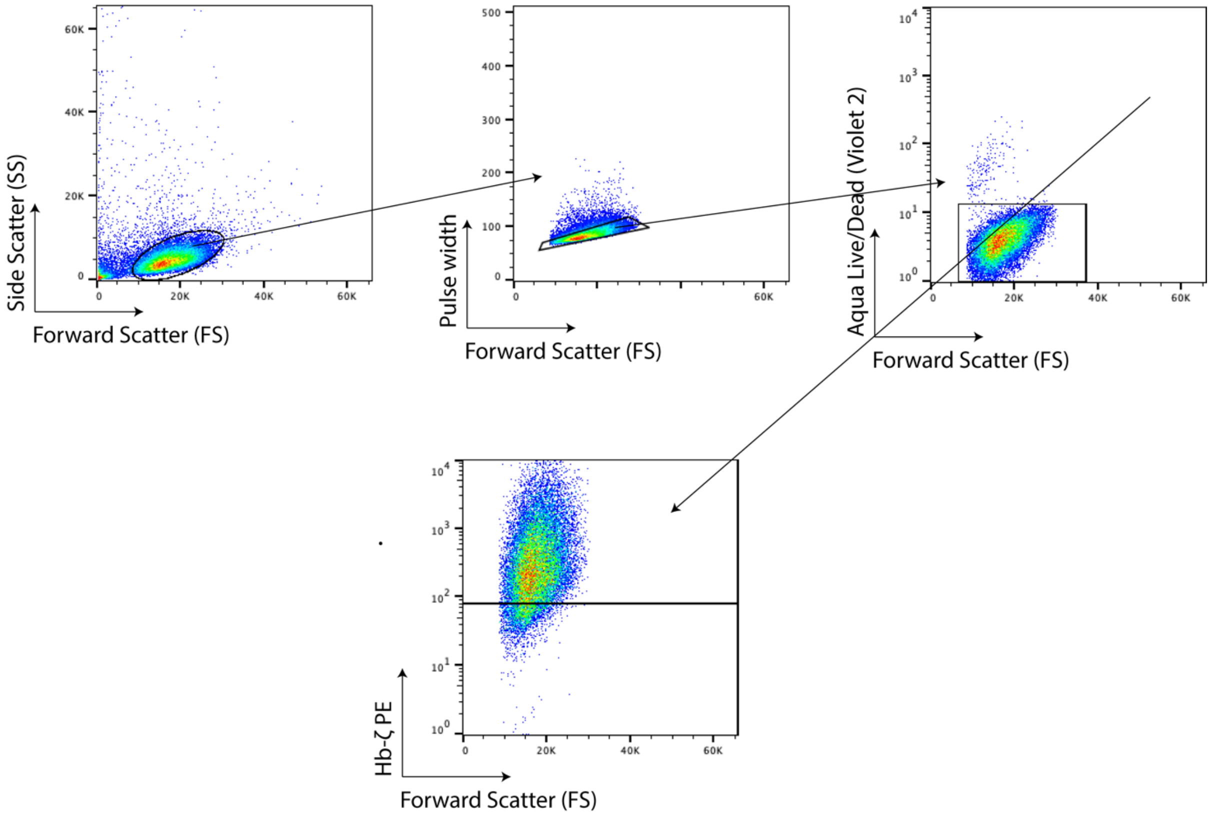

Gating strategy for experiment presented in Figure 7

Supplementary Figure 16

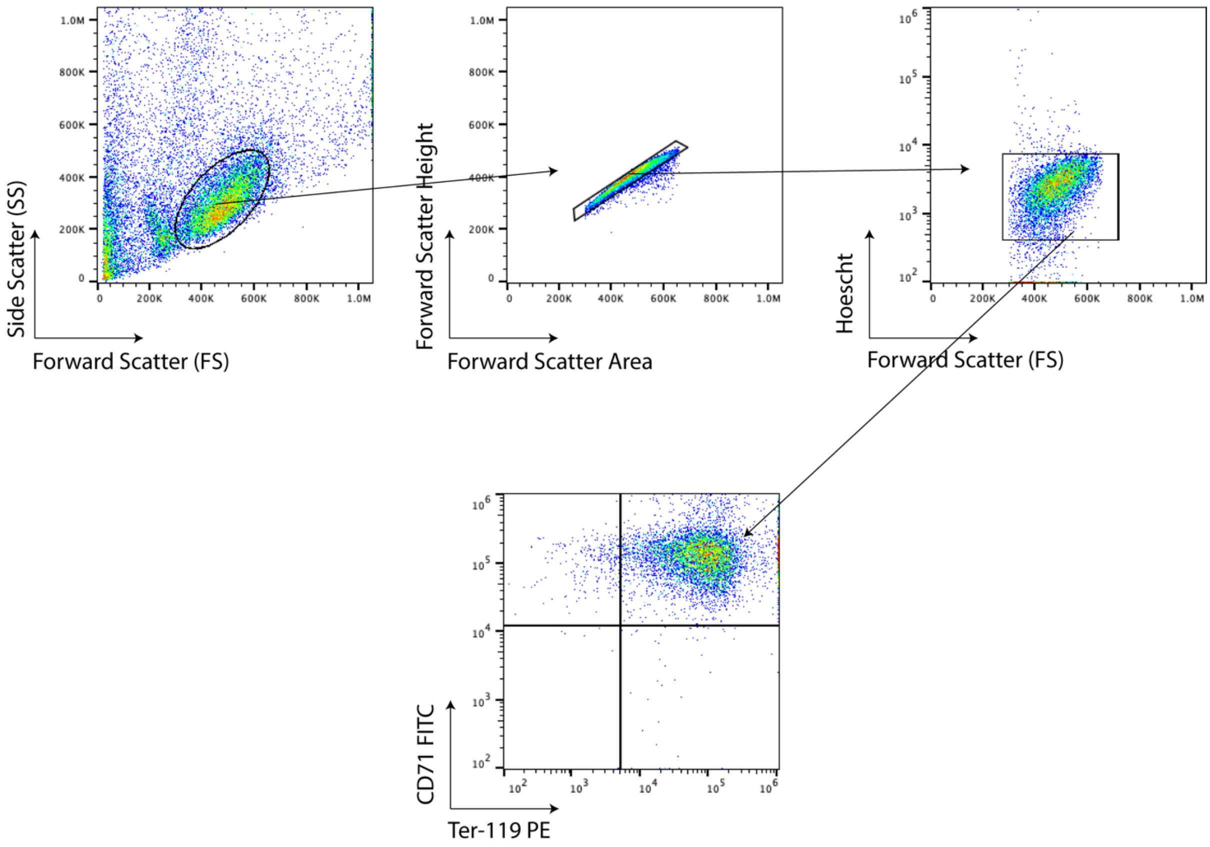

Gating Strategy for experiment presented in Supplementary Figure 1C

Supplementary Figure 17

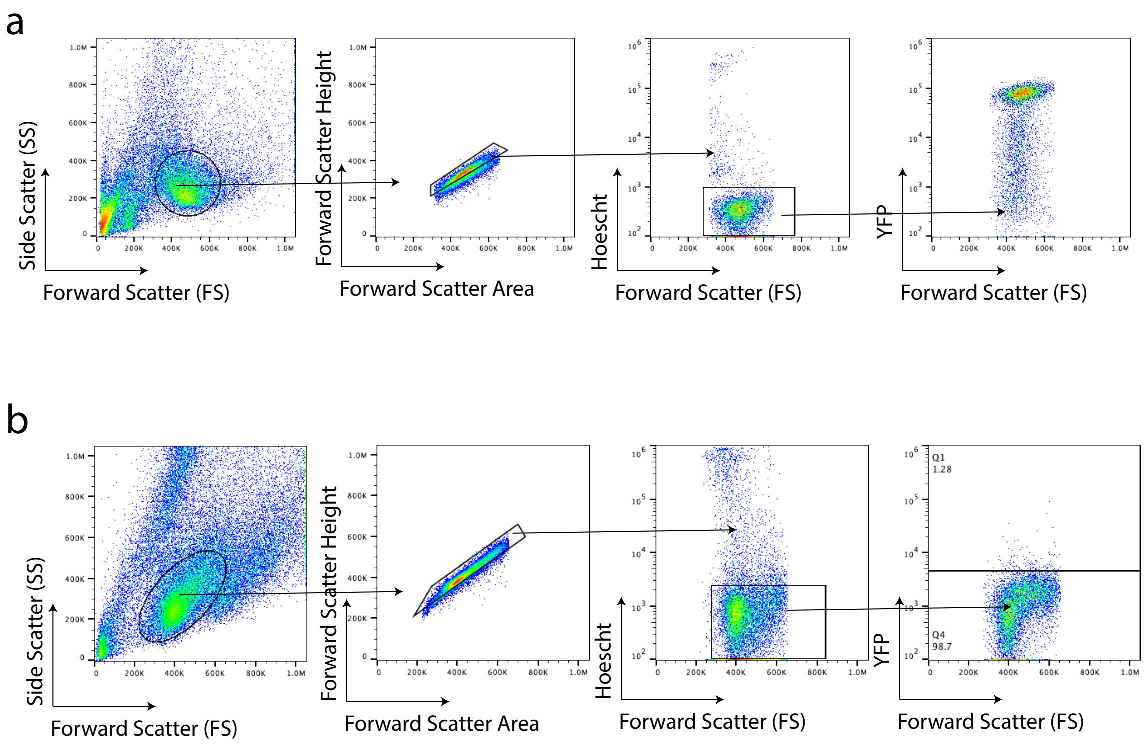

Gating Strategy for experiments presented in Supplementary Figure 6a (a) and Supplementary Figure 6b (b)

Supplementary Figure 18

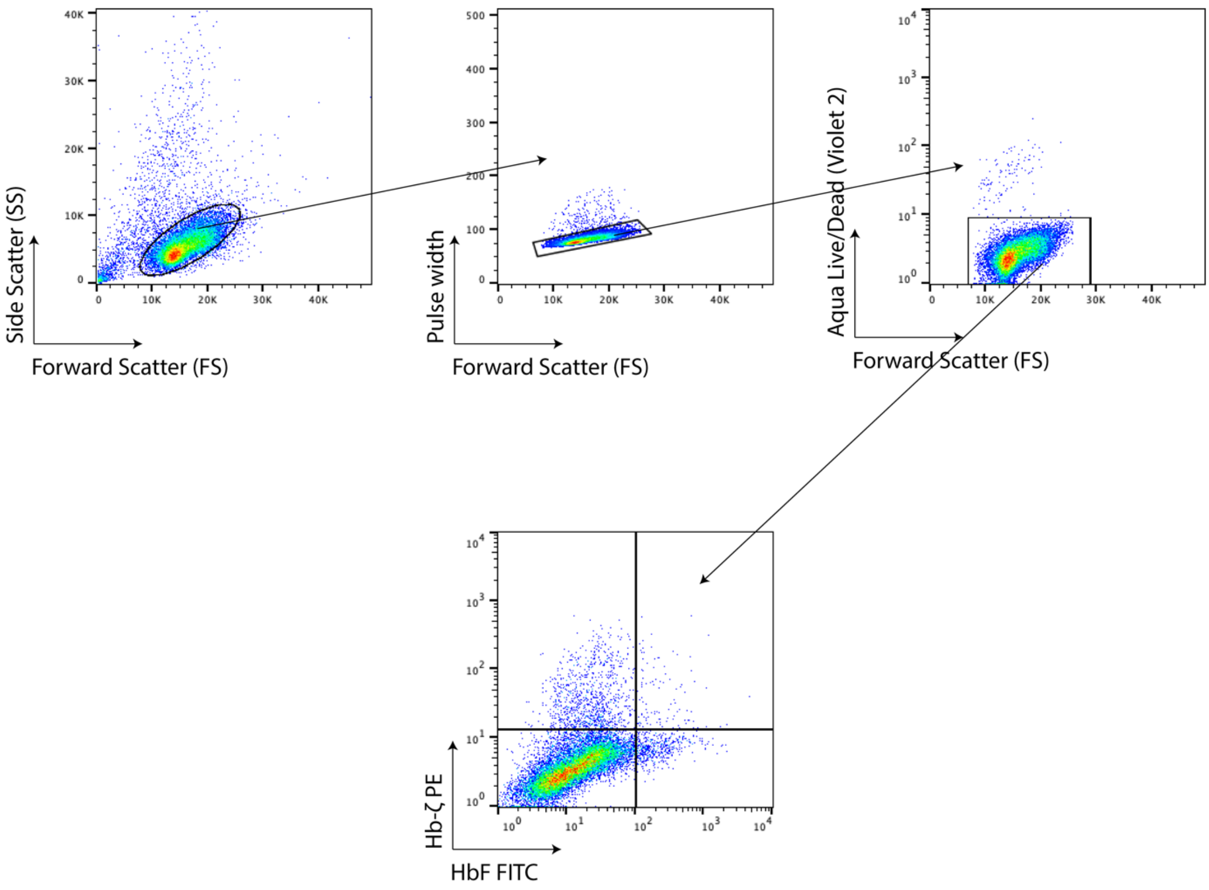

Gating Strategy for experiments presented in Supplementary Figure 10b

Supplementary Table 1

Motifs	JASPAR Predictions
GCGATA	FOXL1=0.64; SPIB=0.62; NFIC=0.53; BRCA1=0.51; GATA3=0.51;
CTGATAAC	GATA3=0.63; SRY=0.6; MZF1_1-4=0.6; NFIC=0.6; FOXL1=0.6; SPIB=0.55; THAP1=0.51; BRCA1=0.51;
GCCCCC	MZF1_1-4=0.78; ZNF354C=0.73; THAP1=0.63; NFIC=0.56; MZF1_5-13=0.55; SP1=0.54; EBF1=0.54; INSM1=0.52;
GCGCAGCGCA	BRCA1=0.72; ZNF354C=0.59; FOXC1=0.59; SPIB=0.52; NRF1=0.51; MZF1_1-4=0.5;
TATCTC	FOXL1=0.69; SPIB=0.63; GATA3=0.63; SRY=0.51;
CCCCCT	MZF1_1-4=0.87; SPIB=0.64; MZF1_5-13=0.63; ZNF354C=0.62; THAP1=0.58; KLF5=0.58; FOXC1=0.57; EBF1=0.54; NFIC=0.53; SP1=0.53; INSM1=0.52; ZEB1=0.51; EHF=0.51;
GTGGGC	ZNF354C=0.9; NFIC=0.71; MZF1_1-4=0.66; THAP1=0.66; ZEB1=0.63; SREBF1=0.56; SREBF2=0.55; MAX=0.54; BRCA1=0.54; SP1=0.53; USF2=0.51;
GGCCCC	MZF1_1-4=0.67; NFIC=0.65; ZNF354C=0.63; THAP1=0.58; EBF1=0.52; KLF5=0.5; REL=0.5;
AATTCC	EHF=0.63; SPIB=0.6; NFIC=0.6; RELA=0.6; REL=0.59; NFKB1=0.55; MZF1_1-4=0.52; DUX4=0.51; ZNF354C=0.51; E2F6=0.51; FOXH1=0.51; E2F4=0.5; HINFP=0.5; NKX3-1=0.5;
ATGGGC	ZNF354C=0.73; NFIC=0.71; MZF1_1-4=0.65; THAP1=0.65; FOXL1=0.57; SREBF2=0.56; SPIB=0.56; SREBF1=0.56; MZF1_5-13=0.55; SP1=0.52; EBF1=0.52; CDX2=0.51; NRF1=0.51;
ATTGGC	NFIC=0.86; THAP1=0.63; SOX9=0.56; BRCA1=0.56; ZNF354C=0.56; FOXL1=0.53; FOXH1=0.52; SPIB=0.52; MZF1_1-4=0.52; DUX4=0.52; CDX2=0.51; SRY=0.5;
GCCCCT	MZF1_1-4=0.77; SPIB=0.64; THAP1=0.62; KLF5=0.59; ZNF354C=0.58; MZF1_5-13=0.58; NFIC=0.56; SP1=0.54; EBF1=0.52; ZEB1=0.52; NRF1=0.51;
TTAGGT	FOXC1=0.64; SPIB=0.64; MZF1_1-4=0.6; NFIC=0.6; ZNF354C=0.59; NKX3-1=0.57; RORA_1=0.55; FOXL1=0.55; THAP1=0.54; DUX4=0.53; SREBF1=0.53; HINFP=0.51; GATA3=0.51;
CCAGCG	BRCA1=0.69; NFIC=0.65; ZNF354C=0.6; FOXC1=0.59; THAP1=0.59; MZF1_1-4=0.58; SPIB=0.54; NRF1=0.51;
GCCCAT	ZNF354C=0.73; NFIC=0.71; MZF1_1-4=0.65; THAP1=0.65; FOXL1=0.57; SREBF2=0.56; SPIB=0.56; SREBF1=0.56; MZF1_5-13=0.55; SP1=0.52; EBF1=0.52; CDX2=0.51; NRF1=0.51;
ATATAAA	FOXL1=0.68; GATA3=0.62; NKX3-1=0.59; FOXD1=0.56; SRY=0.55; FOXP2=0.54; CDX2=0.53; BRCA1=0.52; STAT1=0.51; FOXI1=0.5;
ATACAA	NFIC=0.7; NKX3-1=0.67; FOXL1=0.65; ZNF354C=0.65; SRY=0.65; SOX9=0.61; GATA3=0.61; BRCA1=0.59; FOXD1=0.59; FOXC1=0.58; CDX2=0.5;
GGCTCC	ZNF354C=0.65; NFIC=0.63; BRCA1=0.57; E2F6=0.52; MZF1_1-4=0.52; EBF1=0.52; E2F4=0.51;
TAGAGA	ZNF354C=0.65; SPIB=0.64; FOXL1=0.63; MZF1_1-4=0.61; NFIC=0.52; EBF1=0.52; GATA3=0.51; FOXC1=0.5;
CATGTG	MAX=0.62; NKX3-1=0.59; FOXC1=0.57; USF2=0.56; MZF1_1-4=0.56; BRCA1=0.55; USF1=0.55; ZEB1=0.55; SREBF1=0.53; ZNF354C=0.52; FOXL1=0.52; NRF1=0.52; MYC::MAX=0.51; EBF1=0.51; SPIB=0.51;
CATGTG	MAX=0.62; NKX3-1=0.59; FOXC1=0.57; USF2=0.56; MZF1_1-4=0.56; BRCA1=0.55; USF1=0.55; ZEB1=0.55; SREBF1=0.53; ZNF354C=0.52; FOXL1=0.52; NRF1=0.52; MYC::MAX=0.51; EBF1=0.51; SPIB=0.51;
CCCCTC	MZF1_1-4=0.79; ZNF354C=0.75; SPIB=0.66; MZF1_5-13=0.63; KLF5=0.6; THAP1=0.58; SP1=0.54; FOXC1=0.52; EHF=0.5;
CCCCTC	MZF1_1-4=0.79; ZNF354C=0.75; SPIB=0.66; MZF1_5-13=0.63; KLF5=0.6; THAP1=0.58; SP1=0.54; FOXC1=0.52; EHF=0.5;
CCCCTC	MZF1_1-4=0.79; ZNF354C=0.75; SPIB=0.66; MZF1_5-13=0.63; KLF5=0.6; THAP1=0.58; SP1=0.54; FOXC1=0.52; EHF=0.5;
TACAGT	FOXC1=0.73; SPIB=0.64; FOXL1=0.59; ZNF354C=0.58; SRY=0.56; NFIC=0.55; SOX9=0.53; THAP1=0.51;
ATTCCC	SPIB=0.69; MZF1_1-4=0.69; ZNF354C=0.65; NFIC=0.63; THAP1=0.61; RELA=0.59; REL=0.57; FOXL1=0.56; MZF1_5-13=0.54; NFKB1=0.54; STAT3=0.54; STAT1=0.53; E2F6=0.53; E2F4=0.53; FOXH1=0.52; EHF=0.51;

Footprints at the ζ-globin promoter, sorted by average footprint strength with corresponding JASPAR predictions. Multiple entries represent multiple instances of the same motif identified within the promoter region.

Supplementary Table 2

Element	Genomic coordinates in Mm9	Number of embryos with LacZ activity[(1)]	Staining consistent with erythropoiesis[(2)]
MCSR1	chr11:32145540-32146033 bp	9	Yes
MCSR2	chr11:32150804-32151369	4	Yes
MCSR3	chr11:32156068-32156646	3[(3)]	No
DHS-12	chr11:32165216-32165550	7	No
MCSR4	chr11:32168944-32169344	7	No

Summary of transgenic enhancer testing in E9 mouse embryos.

1. Number of embryos obtained showing any LacZ staining.
2. Enhancer elements were scored as having activity during haematopoiesis if LacZ staining was visualized in hematopoietic cells in at least 3 embryos.
3. A further 6 embryos were shown to carry stable integration of this element.

Supplementary Table 3

Globin	Lineage	Differentiation	α-globin cluster
Hba-a1/a2	Ccr2(Monocyte)	Abcg4	Mpg
Hba-x	Ccr9(Lymphocyte)	Alas2	Nprl3
Hbb-b2	Flt3(Lymphocyte)	αpoe	Rhbdf1
Hbb-bh1	Mpo(Granulocyte)	Arpc2	Sh3pxd2b
Hbb-bh2	Ms4a6c(Monocyte)	Atp1b2	Snrnp25
Hbb-bs	Pf4(Megakaryocyte)	cd59b	
Hbb-y	Plek(Megakaryocye)	ddx21	
Hbq1a	Prtn3 (Granulocyte)	Dek	
Hbq1b	Vwf	Fth1	
Hbb-bt/bs/b1/b2	(Megakaryocye)	Gata2	
	Ifitm1(Basophil)	Gpx1	
	Lmo4(Basophil)	H2afy	
	Nrep(Basophil)	Hnrnpa0	
		Hspe1	
		Klf1	
		Mpl	
		Myc	
		Ncl	
		Nono	
		Sox4	
		Tagln2	
		tmem14c	
		Trib2	
		Vamp5	
		Slc43a3	
		Rhd	
		Podxl	
		Pebp1	

Genes used in single cell analysis of the *Lrf*$^{-/-}$ mice.

Supplementary Table 4

Globin	Lineage	Primitive/Definitive	α-globin cluster
Hba-a2	Ccr2(Monocyte)	Abcg4	Mpg
Hba-x	Mpo(Granulocyte)	Aqp1	Nprl3
Hbb-bh1	Ms4a6c(Monocyte)	Aqp8	Rhbdf1
Hbb-bh2	Prtn3 (Granulocyte)	Arid3a	Snrnp25
Hbb-y	Lmo4(Basophil)	Bcl11a	
Hbq1a		Cited2	
Hbq1b		Cpeb4	
Hbb-bt/bs/b1/b2		Ddx21	
		Foxh1	
		H2afy	
		Hspe1	
		Il9r	
		Klf3	
		Zbtb7a	
		Vamp5	
		Tmem14c	
		Sox6	
		Sox4	
		Smarca4	
		Selk	
		Rhd	
		Pogz	
		Podxl	
		Pbx1	
		Oaz1	
		Myc	
		Mbd2	

Genes used for analysis of $\mathit{Bcl11a}^{\Delta 10kb}$ mice.

Supplementary Table 5

Gene symbol	Target sequence (shown 5′- 3′)	Target description
Alas2	GCCCAACACTTCTCCGAGGCATCTATGGCATCAAAGGATGTTTCTGTTTGGT<br>GTAGTAATGACTATTTGGGCATAAGCAGACACCCTCGTGTCTTGCAGG	Erythroid specific gene
Bod1	ACCAGAACCTAAGGCAGAAGGTGGATAATTTCGTGTCAACACATCTGGACA<br>AACAGGAATGGAATCCTGCAATGAACAAGAACCAGTTGCGAAATGGGCT	Surrounding gene
Btf3	TGGTGCAGACAGCCTGACTAGTTTAAGGAGACTGGCTGAAGCTCTGCCCAA<br>ACAATCTGTGGATGGAAAAGCACCCCTTGCTACTGGAGAGGATGATGAT	Expression control
Car2	TGCCCAGCATGACCCTGCCCTACAGCCTCTGCTCATATCTTATGATAAAGCT<br>GCGTCCAAGAGCATTGTCAACAACGGCCACTCCTTTAACGTTGAGTTT	Expression control
Chac2	GTGTATGGGGTGTTGCTTACAAACTACCAGTAGGAAAAGAAGAGGAAGTA<br>AAAACATACCTTGACTTCAGAGAGAAAGGAGGCTACAGAACTACGACAGT	Surrounding gene
Cpeb4	GCATCGTCCCTTACTGGTTTCAGTAACTGGTCAGCAGCGATAGCACCTTCTT<br>CCTCCACTATAATCAATGAAGATGCAAGTTTCTTTCACCAGGGAGGGG	Surrounding gene
Eef2	TGGACCAGTTCCTTGTGAAGACGGGGACCATCACTACCTTTGAGCACGCTC<br>ACAACATGCGCGTGATGAAGTTCAGCGTCAGCCCTGTCGTCCGTGTGGC	Expression control
Fth1	TGGGAGAGCGGGCTGAATGCAATGGAGTGTGCACTGCACTTGGAAAAGA<br>GTGTGAATCAGTCACTACTGGAACTGCACAAACTGGCTACTGACAAGAATG	Erythroid specific gene
Gypa	GGCTTCAACTGTAGGTAACCCAAATCAGCATTCAGCAACAATGTCAACACC<br>AGCTATTCATGTCTCAACTTATCACACGGCCCCTACTGAAGTGTCTGCT	Erythroid specific gene
Hba-a1	GAAGAAACCATGGTGCTCTCTGGGGAAGACAAAAGCAACATCAAGGCTGC<br>CTGGGGGAAGATTGGTGGCCATGGTGCTGAATATGGAGCTGAAGCCCTGG	Globin gene
Hba-x	CTGGTCACAATGGCCGCACGCTTTCCCGCCGACTTCACCCCTGAGGTCCAC<br>GAAGCCTGGGACAAGTTCATGTCTATCCTGTCTTCTATCCTGACTGAGA	Globin gene
Hbb-bh1	CACAAGTACCATTAAACTCCATTCCAGTACACTGGCAATCCCATGTGTCTAT<br>GATGCCCTCTTTGAGTCCATGGGGACTGCATTGAGAGCACAAATTTTG	Globin gene
Hbb-bh2	GGTGTTGAGTCCCTGGAAAGAATACTGACGGTTTATCCACACACCAAGAGA<br>TACTTTGACCACTTTGGAGACTTCTCCTTCTGTGCTGCCACTGAAGATA	Globin gene
Hbb-b1	ACCTATCCTCTGCCTCTGCTATCATGGGTAATGCCAAAGTGAAGGCCCATG<br>GCAAGAAAGTGATAACTGCCTTTAACGATGGCCTGAATCACTTGGACAG	Globin gene
Hbb-y	GTACCACTGAGCCCTCTCTCTAGCTGTCCAGCAATCCTGTGTGTCCGCTATG<br>CCTCCTTTCTGCACATGAATACTGGACTGTTCCTTGAAAGCACATCAT	Globin gene
Hbq	TCACGTGACTTCGGCACTGGTCTCCAAATATCGCTGAATTGGGGATACAGG<br>GTTCTATAGTCTCCACCCATGCCCTTCCTAAAGCTCACAATGTTTGAGT	Globin gene
Il9r	TGACTGAAATCAAACACAAATGCACCTTCTGGGACAGTATGTGTACCCTGG<br>TGCTGCCTAAAGAGGAGGTGTTCTTACCTTTTGACAACTTCACCATCAC	Surrounding gene
Mpg	AAAGAGAGACTCCTGTCGACCCCGGGCCTCCGGCGGAGTATCTACTTCTCC<br>AGCCCAGAGGACCATTCTGGCCGGCTAGGACCAGAGTTTTTTGACCAGC	Surrounding gene
Npm1	CTGTTCTGTGGAACAGGAGGCAGTTGTTTTCCGTCCGGCTTCTCCCACACCG<br>AAGTGCGCGCCTCCACCTCATGGAAGACTCGATGGATATGGACATGAG	Surrounding gene
Nprl3	TCCACTATGCAGCTTCAAGTCTGATTCCTCCTGAGGCTATTGAACGGAGCCT<br>GAAAGCCATCCGCCCGTACCATGCCTTGCTACTTCTCAGTGACGAGAA	Surrounding gene
Pabpc1	TGATGAAAATGGCTCCAAGGGCTATGGATTTGTACACTTTGAAACACAGGA<br>AGCAGCTGAAAGAGCTATTGAAAAAATGAATGGGATGCTTCTAAATGAT	Expression control
Paip2	AAGTCGCAGCAGTACTAGCCCAAGCATCATCAATGACGATGTGATTATTAA<br>CGGTCATTCTCATGAAGAGGATAATCCATTTGCAGAGTACATGTGGATG	Expression control
Rhbdf1	GGGTGCTTCCTGGACACTGACCTCTTGTGCCTTGCTCGCTCCGGTTGTACGC<br>TTACACTGCTGGGCATTTAGTACATGAGTTCCCATGATAACCTCCTAA	Surrounding gene
Rpl38	GAGGAGATCAAGGACTTTCTGCTGACAGCCCGGCGGAAGGATGCCAAGTC<br>TGTCAAGATCAAGAAGAACAAGGATAATGTGAAGTTCAAGGTTCGCTGCA	Expression control
Rps18	CAGCACACCAAGACCACTGGCCGCAGGGGCCGAACTGTGGGTGTATCCAA<br>GAAGAAATGAGTCTCTGGGCCTTTGCTGTTAATAAATAGTTTATATACCT	Expression control
Sh3pxd2b	ATGGTCCTGGAGCAGTATGTGGTAGTGGCAGACTATCAGAAGCAGGAGAG<br>CTCAGAGATCAGCCTCAGTGTGGGCCAAGTAGTGGACATCATTGAGAAGA	Surrounding gene
Shmt2	CCGACTTGTTTTTGAGACGGGGGTCTCACTTAGCTGTGAGTGGCCTTGAAC<br>TGACCCTTCCACCTTCATTTCCCAAGTGCTGGGGTTATAGGTTTGCACC	Expression control
Slc4a1	GGCAGCGCACTGGATAGGGCTGGAGGAAAACCTTCGAGAGGATGGTGTA<br>TGGGGTCGCCCACATCTATCTTACCTGACCTTCTGGAGCCTTCTAGAACTG	Erythroid specific gene
Snrnp25	CGGAGGACGACGAGGATGATGAGGAGACATTGCCCCATTCCGAGGCTGTG<br>GACGTGTTCCAAGAAGGTCTCGCCATGGTGGTGCAAGACCCGCTGCTCTG	Surrounding gene
Stk10	TGTCGTCGCGCTCCTCATCCAGCATGGCTTTCGCCAATTTCCGCCGCATCTT<br>GCGGTTATCCACCTTCGAGAAGAGAAAGTCCCGTGAATATGAGCACGT	Surrounding gene
Ubtd2	CGTAACCAGCCTTTGAAAAAGGAGAAACCAAAATGGAAAAGTGACTATCC<br>CATGACAGATGGACAACTGCGCAGCAAGAGGGATGAATTCTGGGACACTG	Surrounding gene

Capture probes included in the nCounter® expression assay.

Supplementary Table 6

Sequence 5' to 3'
ACGTAAACGGCCACAAGTTC
CGTTGTGGCTGTTGTAGTTG
TCTGATAACAGGCTCCACC
TAAACCATGGGATCGGCCATTG
ATTCAGCCTCTAACTCTACTTGC
ACAGGGATCTGCCATGTAGAC
GAAGCTAGAGGCATCAAATC
CTTCCATTGCTCAGCGGTG
CGTGCAATCCATCTTGTTCAATG
CAGCTTGCCGTAGGTGGCATC
GACGGCGAGGATCTCGTCGTGAC
CAATACGCCCGCGTTTCTTCC
GTCCCTGTCCCTAAAGGATAAG
ATCCGGACCACCCATGTGCAG
CCATGTGCAGATTTTACAAGCTC
GGCATCGACTTCAAGGAGGA
CGAAGCGATTTGGGAAGGTG

Sequencing primers used during generation of *Hba-x*-Venus Mouse.

Supplementary Table 7

Target	Catalogue Number
**HUMAN**	
HBA (HBA1/2)	Hs00361191_g1
HBB	Hs00747223_g1
HBZ	Hs00923579_m1
HBE	Hs00362216_m1
HBG (HBG1/2)	Hs00361131_g1
RPL13A	Hs04194366_g1
**MOUSE**	
Hba-a (Hba-1/2)	Mm02580841_g1
Hbb-b	Mm01611268_g1
Hba-x	Mm00439255_m1
Hbb-y	Mm00433936_g1
Hbb-bh1	Mm00433932_g1
Bcl11a	Mm00479358_m1
Rps18	Mm02601777_g1

TaqMan probes used for RT-qPCR analysis. Note that the *HBA* and *Hba-a* probe targets both α-globin genes; similarly, the *HBG* probe targets both human γ-globin genes.

Supplementary Table 8

Target	Sequence
Hba-x	F ATGCGGTTAAGAGCATCGAC
	R GGGACAGGAGCTTGAAGTTG
Venus	F CAAAGACCCCAACGAGAAGC
	R CTTGTACAGCTCGTCCATGC

Custom primers (Sigma-Aldrich) used for RT-qPCR analysis.

Supplementary Table 9

Target	Catalogue Number	Target	Catalogue Number
Abcg4	Mm00507247_m1	*Pbx1*	Mm04207617_m1
Aqp1	Mm01326466_m1	*Podxl*	Mm00449829_m1
Aqp8	Mm00431846_m1	*Pogz*	Mm00554475_m1
Arid3a	Mm00492248_m1	*Prtn3*	Mm00478323_m1
Ccr2	Mm00438270_m1	*Rhbdf1*	Mm00711711_m1
Cited2	Mm00516121_m1	*Rhd*	Mm00456910_m1
Cox6c	Mm00835813_g1	*Sec61g*	Mm01613099_g1
Cpeb4	Mm01193440_m1	*Selk*	Mm00785961_s1
Ddx21	Mm00497941_m1	*Smarca4*	Mm01151944_m1
Foxh1	Mm00514851_m1	*Snrnp25*	Mm00547218_m1
H2afy	Mm01337623_gH	*Sox4*	Mm00486320_s1
Hba-a1/2	Custom (IDT)	*Sox6*	Mm01274768_m1
Hba-x	Mm00439255_m1	*Tmem14c*	Mm00481276_m1
Hbb-bh1	Mm00433932_g1	*Vamp5*	Mm00444144_m1
Hbb-bh2	Mm01273444_g1	*Zbtb7a*	Mm00657132_m1
Hbb-bt/bs/b1/b2	Custom (IDT)	*Hbb-b2*	Mm00731743_mH
Hbb-y	Mm00433936_g1	*Ccr9*	Mm02528165_s1
Hbq1a	Mm00731011_s1	*Flt3*	Mm00439016_m1
Hbq1b	Mm02747875_s1	*Ms4a6c*	Mm00459296_m1
Hspe1	Mm00434083_m1	*Pf4*	Mm00451315_g1
Il9r	Mm00434313_m1	*Plek*	Mm00450970_m1
Klf3	Mm00492956_m1	*Ifitm1*	Mm00850040_g1
Lmo4	Mm00495373_m1	*Nrep*	Mm00474047_m1
Mbd2	Mm00521967_m1	*Vwf*	Mm00550376_m1
Mpg	Mm00447872_m1	*Alas2*	Mm00802083_m1
Mpo	Mm01298424_m1	*ApoE*	Mm01307193_g1
Myc	Mm00487804_m1	*Arpc2*	Mm01254383_m1
Myl6	Mm02342525_g1	*Arpc2*	Mm01254383_m1
Nprl3	Mm01193449_m1	*Atp1b2*	Mm00442612_m1
Oaz1	Mm01611061_g1	*CD59b*	Mm02525679_s1
Ddx21	Mm00497941_m1	*Dek*	Mm01351566_m1
Fth1	Mm00850707_g1	*Hnrnpa0*	Mm03809085_s1
Gata2	Mm00492301_m1	*Klf1*	Mm00516096_m1
Gpx1	Mm00656767_g1	*Mpl*	Mm00440310_m1
Ncl	Mm01290600_g1	*Nono*	Mm00834875_g1
Tagln2	Mm00724260_g1	*Slc43a3*	Mm00469627_m1
Trib2	Mm00454876_m1	*Pebp1*	Mm02601848_g1
Sh3pxd2b	Mm00616672_m1	*Hbb-b1*	Mm03646870_gH

Taqman assays used in single cell experiment.

Supplementary Table 10

Target	Left Primer	Right Primer	Internal Oligo
Hba-a1/a2	TGAAGCCCTGGAAA GGATGT	TGAAATCGGCAGGG TGGT	TGGATCCCGTCAACTTCA AGCTCCT
Hbb-bt/bs/b1/b2	CCGATGAAGTTGGT GGTGAG	ACATGCAGCTTGTC ACAGTG	CCTGGGCAGGCTGCTGG TTG

Custom Taqman assays used in single cell experiment.

Supplementary Table 11

Target		Sequence 5’ to 3’	Sequence 5’ to 3’
HBZ promoter and gene	1A	F GGAAAGTAATATTGTAATGT	R AAATCTAACCACAAAATCTA
	1B	F GTATATGATATTAGGTGAAT	R ATCACTCCTACATTTATAATC
	2A	F GTTGAGTATTATTAAGGTTAG	R CTATCCTCTCCCCACAATAC
	2B	F GTAGGTTTAATTTTAGTGTAG	R CACTATCTCCTCTCCATAATC
	3A	F GATTATGGAGAGGAGATAGTG	R CAATACCCTAATCCCAAATAC
	3B	F GTATTGTGGGGAGAGGATAG	R AACTATAATACCCTAACTAC
	4A	F GTATTTGGGATTAGGGTATTG	R CCTCCCAAACTACCCTAAAC
	4B	F GTATTTGATTTTTTGGGAGG	R ACCCCCCAAACCTCTACAAC
	5A	F GTTGTAGAGGTTTGGGGGGT	R AAAAATAAATCTTAATCTA
	5B	F GTTTAGGGTAGTTTGGGAGG	R AAAAATAAATCTTAATCTA
	6A	F AGTTTTTGTTTTATTGTTTG	R ACTACACATCCCTCTACCTC
	6B	F GTTTTATTGTTTGTTGGTTATTTTG	R CTACCTCTTAAAAACCCTAA
	7A	F TTTATTTGGTTTTTGTTGTT	F CATAACCTAAAAACATACCT
	7B	F GAGGTAGAGGGATGTGTAGT	R CTTATACCCAATAAATATTTC
	8A	F GAAATATTTATTGGGTATAAG	R CAATTAATCCTAATAATAAAC
	8B	F AGGTATGTTTTTAGGTTATG	R CTAAATTTTCAAATCTTTAAC
ACTB	1A	F GAGGGGGTTGGGGTTTGG	R CTACCCCACCCAACCAACT
	1B	F GTTTGTTTTTTATGGTAATAA	R CTCCCCTACCTAATACCTA

Supplementary Table 11 Nested primers used for Bisulfite sequencing. A denotes the outside primer set and B, the internal primer set.

Supplementary Table 12

Figure	Cell Type	Data Type	GEO Code	Publication
Figure 3	Ter119 + Splenic erythroblasts (definitive)	H3K4me1 ChIP-seq H3K27ac ChIP-seq	GSE27921	Reference 9
Figure 3	Ter119 + Splenic erythroblasts (definitive)	CTCF ChIP-seq H3K27me3 ChIP-seq H3K4me3 ChIP-seq	GSE97871	Reference 6
Supplementary Figure 5	WT HUDEP-2	LRF ChIP-seq	GSE74977	Reference 10
Supplementary Figure 5	HUDEP-2 BCL11A-ER-V5	BCL11A ChIP-seq	GSE103445	Reference 3
Supplementary Figure 5	HUDEP-2 (ΔGγ)	LRF ChIP-seq	GSE103445	Reference 3
Supplementary Figure 5	WT HUDEP-2	BCL11A CUT&RUN	GSE104676	Reference 4
Supplementary Figure 5	Primary human adult proerythroblasts from $CD34^+$ culture	H3K9me3 ChIP-seq H3K27me3 ChIP-seq	GSE36994	Reference 2
Supplementary Figure 5	Primary human adult erythroid cells from CD34+ culture	RNA-seq adult cells	GSE71422	Reference 5

Previously published data used in this manuscript.

References

1. Hubisz, M. J., Pollard, K. S. & Siepel, A. PHAST and RPHAST: phylogenetic analysis with space/time models. *Brief Bioinform* **12**, 41-51, doi:10.1093/bib/bbq072 (2011).

2. Xu, J., Shao, Z., Glass, K., Bauer, D. E., Pinello, L., Van Handel, B., et al. (2012). Combinatorial assembly of developmental stage-specific enhancers controls gene expression programs during human erythropoiesis. Developmental Cell, 23(4), 796–811

3. Martyn, G.E., Wienert, B., Yang, L., Shah, M., Norton, L.J., Burdach, J., Kurita, R., Nakamura, Y., Pearson, R.C.M., Funnell, A.P.W., et al. (2018). Natural regulatory mutations elevate the fetal globin gene via disruption of BCL11A or ZBTB7A binding. Nature Genetics *50*, 498–503.

4. Liu, N., Hargreaves, V.V., Zhu, Q., Kurland, J.V., Hong, J., Kim, W., Sher, F., Macias-Trevino, C., Rogers, J.M., Kurita, R., et al. (2018). Direct Promoter Repression by BCL11A Controls the Fetal to Adult Hemoglobin Switch. Cell *173*, 430–442.e17.

5. Renneville, A., Van Galen, P., Canver, M. C., McConkey, M., Krill-Burger, J. M., Dorfman, D. M., et al. (2015). EHMT1 and EHMT2 inhibition induces fetal hemoglobin expression. Blood, 126(16), 1930–1939.

6. Hanssen, L.L.P., Kassouf, M.T., Oudelaar, A.M., Biggs, D., Preece, C., Downes, D.J., Gosden, M., Sharpe, J.A., Sloane-Stanley, J.A., Hughes, J.R., et al. (2017). Tissue-specific CTCF-cohesin-mediated chromatin architecture delimits enhancer interactions and function in vivo. Nat. Cell Biol. *19*, 952–961.

7. Li, T. K. T., Leung, K. Y., Lam, Y. H., Tang, M. H. Y., & Chan, V. (2010). Haemoglobin level, proportion of haemoglobin Bart"s and haemoglobin Portland in fetuses affected by homozygous α0-thalassemia from 12 to 40 weeks" gestation. *Prenatal Diagnosis*, *30*(12-13), 1126–1130.

8. Viprakasit, V., Ekwattanakit, S., Riolueang, S., Chalaow, N., Fisher, C., Lower, K., et al. (2014). Mutations in Kruppel-like factor 1 cause transfusion-dependent hemolytic anemia and persistence of embryonic globin gene expression. Blood, *123*, 1586–1595.

9. Kowalczyk, M.S., Hughes, J.R., Garrick, D., Lynch, M.D., Sharpe, J.A., Sloane-Stanley, J.A., McGowan, S.J., De Gobbi, M., Hosseini, M., Vernimmen, D., et al. (2012). Intragenic enhancers act as alternative promoters. Mol. Cell *45*, 447–458

10. Masuda, T., Wang, X., Maeda, M., Canver, M.C., Sher, F., Funnell, A.P.W., Fisher, C., Suciu, M., Martyn, G.E., Norton, L.J., et al. (2016). Transcription factors LRF and BCL11A independently repress expression of fetal hemoglobin. Science *351*, 285–289.
