# Supplementary material for: Reactivation of a developmentally silenced embryonic globin gene
Source: Nat Commun. 2021 Jul 21;12:4439. doi: 10.1038/s41467-021-24402-3 (PMC8295333; doi:10.1038/s41467-021-24402-3)
Supplement: Supplementary file 2 — Reporting Summary [file 41467_2021_24402_MOESM2_ESM.pdf]

nature research

Corresponding author(s): *Double-blind peer review submissions: write DBPR and your manuscript number here instead of author names.*

Last updated by author(s): *YYYY-MM-DD*

Reporting Summary

Nature Research wishes to improve the reproducibility of the work that we publish. This form provides structure for consistency and transparency in reporting. For further information on Nature Research policies, see our Editorial Policies and the Editorial Policy Checklist.

Statistics

For all statistical analyses, confirm that the following items are present in the figure legend, table legend, main text, or Methods section.

n/a	Confirmed	
☐	☒	The exact sample size (*n*) for each experimental group/condition, given as a discrete number and unit of measurement
☒	☐	A statement on whether measurements were taken from distinct samples or whether the same sample was measured repeatedly
☐	☒	The statistical test(s) used AND whether they are one- or two-sided<br>*Only common tests should be described solely by name; describe more complex techniques in the Methods section.*
☒	☐	A description of all covariates tested
☒	☐	A description of any assumptions or corrections, such as tests of normality and adjustment for multiple comparisons
☐	☒	A full description of the statistical parameters including central tendency (e.g. means) or other basic estimates (e.g. regression coefficient) AND variation (e.g. standard deviation) or associated estimates of uncertainty (e.g. confidence intervals)
☐	☒	For null hypothesis testing, the test statistic (e.g. *F*, *t*, *r*) with confidence intervals, effect sizes, degrees of freedom and *P* value noted<br>*Give P values as exact values whenever suitable.*
☒	☐	For Bayesian analysis, information on the choice of priors and Markov chain Monte Carlo settings
☒	☐	For hierarchical and complex designs, identification of the appropriate level for tests and full reporting of outcomes
☒	☐	Estimates of effect sizes (e.g. Cohen's *d*, Pearson's *r*), indicating how they were calculated

Our web collection on statistics for biologists contains articles on many of the points above.

Software and code

Policy information about availability of computer code

Data collection	No Software was used for data collection in this study
Data analysis	ChIP-seq and ATAC-seq data were aligned to the mm9 (for mouse) and hg19 (for human) data using a customised in house pipeline (script: https://github.com/Hughes-Genome-Group/NGseqBasic/releases). Carture Data were analyzed using analysis scripts (github.com/Hughes-Genome-Group/CCseqBasicF), and custom perl and R scripts were used to normalize data and generate differential tracks (github.com/djdownes/CaptureCompare). The following software packages were used for genomics analysis: Bedtools v2.25.0, ROSE tool v1.0, Samtools v0.1.19, MACS v2.0.10, UCSC tools v1.0, SeqMonk v0.24.1, STAR v2.4.2a, Deeptools v2.2.2, FASTQC v0.11.4. For Micro Capture-C data were analysed with a custom analysis pipeline specifically developed for MCC data analysis as detailed in Hua et al, 2021. DNAse footprinting in Supplementary Figure 2 and Supplementary Table 1 was generated using PyDNAse Python package v0.1.7. Flow cytometry analysis was performed with FlowJo v10.3.

For manuscripts utilizing custom algorithms or software that are central to the research but not yet described in published literature, software must be made available to editors and reviewers. We strongly encourage code deposition in a community repository (e.g. GitHub). See the Nature Research guidelines for submitting code & software for further information.

Data

Policy information about availability of data

All manuscripts must include a data availability statement. This statement should provide the following information, where applicable:

- Accession codes, unique identifiers, or web links for publicly available datasets
- A list of figures that have associated raw data
- A description of any restrictions on data availability

Source data availability: Raw sequence data generated are available on the Gene Expression Omnibus (GEO) database under the following accessions: GSE108434

(Open chromatin, Capture-C and epigenetic data in wild type primitive murine cells), GSE174110 (Open chromatin data in mouse models), GSE153256 (Micro Capture-C in definitive murine erythroid cells), and GSE173419 (Open chromatin and epigenetic data in edited HUDEP2 cells). These data are also available in the following UCSC datahub: https://datashare.molbiol.ox.ac.uk/public/project/fgenomics/publications/King_2021_Zeta_Activation/hub.txt . Source Data for all figures are provided in the Source Data file. All other data supporting the findings of this study are available from the corresponding author on request. Published datasets analysed (see Supplementary Table 12 for details): GSE137477, GSE27921, GSE97871, GSE 74977, GSE103445, GSE 104676, GSE36944, GSE 71422.

Field-specific reporting

Please select the one below that is the best fit for your research. If you are not sure, read the appropriate sections before making your selection.

[x] Life sciences   [ ] Behavioural & social sciences   [ ] Ecological, evolutionary & environmental sciences

For a reference copy of the document with all sections, see nature.com/documents/nr-reporting-summary-flat.pdf

Life sciences study design

All studies must disclose on these points even when the disclosure is negative.

Sample size	Sample sizes were selected with a minimum of N=3 to ensure reproducibility and allow descriptive statistics to be performed. In Figure 2B a sample size of 3 was used to allow statistical significance to be calculated using a one way ANOVA with Dunnett correction for multiple comparisons.
Data exclusions	No data were excluded.
Replication	All findings were replicated in a minimum of three experiments, all attempts at replication were successful.
Randomization	Experimental groups of mice and cells were determined by genotype natural, catergorical or inhereent.
Blinding	Blinding was not relevant to this study as the genomics data presented here are objectively obtained by quantification of cellular phenotypes through measurements of mRNA levels, protein levels and chromatin interactions. Experimental groups were selected by genotype.

Reporting for specific materials, systems and methods

We require information from authors about some types of materials, experimental systems and methods used in many studies. Here, indicate whether each material, system or method listed is relevant to your study. If you are not sure if a list item applies to your research, read the appropriate section before selecting a response.

Materials & experimental systems

n/a	Involved in the study	
[ ]	[x]	Antibodies
[ ]	[x]	Eukaryotic cell lines
[x]	[ ]	Palaeontology and archaeology
[ ]	[x]	Animals and other organisms
[x]	[ ]	Human research participants
[x]	[ ]	Clinical data
[x]	[ ]	Dual use research of concern

Methods

n/a	Involved in the study	
[ ]	[ ]	ChIP-seq
[ ]	[ ]	Flow cytometry
[ ]	[ ]	MRI-based neuroimaging

Antibodies

Antibodies used	anti-CD44; Supplier: BD Biosciences, Catalog Number: 561862 Clone Name IM7 (https://www.bdbiosciences.com/eu/applications/research/t-cell-immunology/t-follicular-helper-tfh-cells/surface-markers/mouse/apc-rat-anti-mouse-cd44-im7/p/559250)<br>anti-Ter119; Supplier: BD Biosciences, Catalog Number: 561071 Clone Name TER119 (https://www.bdbiosciences.com/eu/applications/research/stem-cell-research/mesoderm-markers/mouse/pe-rat-anti-mouse-ter-119erythroid-cells-ter-119/p/553673)<br>anti-CD71; Supplier: BioLegend, Catalog Number: 113811 Clone Name: RI7217 (ttps://www.biolegend.com/en-us/products/pe-cyanine7-anti-mouse-cd71-antibody-6185)<br>PE Mouse anti-human CD235a:Supplier BD Biosciences; Catolog number 555570;Clone Name GA-R2 (HIR2) (https://www.bdbiosciences.com/us/reagents/research/antibodies-buffers/immunology-reagents/anti-human-antibodies/cell-surface-antigens/pe-mouse-anti-human-cd235a-ga-r2-hir2/p/555570)<br>FITC mouse anti-human Band 3;Supplier IBGRL Research Products; Product Code 9439; Clone name BRIC 6 (https://nhsbtdbe.blob.core.windows.net/umbraco-assets-corp/6596/bric-6.pdf)<br>APC- Mouse antiCD49d; Supplier BD Biosciences; Catalogue number 559881; Clone name: 9F10 (https://www.bdbiosciences.com/us/applications/research/stem-cell-research/mesenchymal-stem-cell-markers-bone-marrow/human/positive-markers/apc--mouse-anti-human-cd49d-9f10/p/559881)<br>PE/Cy7 anti CD34: Supplier Biolegend; Catalogue number 343616; Clone name: 561 (https://www.biolegend.com/en-us/products/

pe-cyanine7-anti-human-cd34-antibody-13588)
APC/Cy7 anti CD36 (BioLegend 1:100); Supplier Biolegend; Catalogue number 336213: Clone name: 5-271 (https://www.biolegend.com/en-us/products/apc-cyanine7-anti-human-cd36-antibody-5457)
anti-zeta-globin:As reported in Luo, H.Y., Clarke, B.J., Gauldie, J., Patterson, M., Liao, S.K., and Chui, D.H. (1988). A novel monoclonal antibody based diagnostic test for alpha-thalassemia-1 carriers due to the (-SEA/) deletion. Blood 72, 1589–1594.

Validation

All antibodies (apart from zeta-globin primary antibody, see below) were obtained from commercial suppliers and validated by the vendor for the species and assay used in the studies presented here (see websites listed above).

To verify the zeta globin antibody was specific to zeta-globin as opposed to other globins using flow cytometry, two experiments were performed. Firstly, blood from a Bart's Hydrops terminated fetus at 18 weeks (obtained with appropriate consent) and adult blood from a healthy donor was subject to flow cytometry using the antibody. A strong signal was seen in the terminated fetus as opposed to adult blood. This excluded cross reactivity with !-globin, beta-globin and epsilon-globin (which has been silenced by 18 weeks). To exclude crossreactivity with gamma-globin, WT HUDEP-2 cells, were FACS sorted after staining. This showed negligible amounts of gamma-globinin the zeta-globin positive fraction. This confirmed that the zeta-globin antibody is specific.

Eukaryotic cell lines

Policy information about cell lines

Cell line source(s)

WT HUDEP2 and Double KO HUDEP2 cells were obtained from the laboratory of Daniel Bauer, 1 Division of Hematology/Oncology, Boston Children's Hospital, Department of Pediatric Oncology, Dana-Farber Cancer Institute, Harvard Stem Cell Institute, Broad Institute, Department of Pediatrics, Harvard Medical School, Boston, MA, USA. E14 mouse embryonic stem cells were derived in house.

Authentication

WT and mutant HUDEP2 cell lines were authenticated by aCGH analysis. Mouse ES cells were characterised by sequencing of the alpha-globin locus and mVenus integration site and karyotyping.

Mycoplasma contamination

Cells were negative of mycoplasma contamination using the MycoAlert kit from Lonza.

Commonly misidentified lines
(See ICLAC register)

None

Animals and other organisms

Policy information about studies involving animals; ARRIVE guidelines recommended for reporting animal research

Laboratory animals

Mice, wild type mice were C15Bl6/J WT mice and Hba-x-mVenus transagenic mice on a C15Bl6J/129sv/ev mixed strain background, both genders. Ages ranged from embryonic day 8.5 to six months of age.

Wild animals

No wild animals were used in this study

Field-collected samples

No field collected samples were used in this study.

Ethics oversight

This work was conducted following ethical review by the Medical Sciences Local Ethical Review Panel of the University of Oxford. The work was regulated by the United Kingdom Home Office (licence numbers PPl 30/3328 and PP1728514)

Note that full information on the approval of the study protocol must also be provided in the manuscript.

ChIP-seq

Data deposition

☒ Confirm that both raw and final processed data have been deposited in a public database such as GEO.

☒ Confirm that you have deposited or provided access to graph files (e.g. BED files) for the called peaks.

Data access links
May remain private before publication.

Accession codes. ATAC-seq and NG Capture-C data are available under Gene Expression Omnibus (GEO) accession GSE108434 with token code ihefoqguvzybfal and Tiled-C data under accession number GSE137477. Micro Capture-C data are available under GEO GSE153256 with token code kdsnguguvzkftex. NG Capture-C data from the enhancers and CTCF at the Hba locus in ESC is from GSE97871. All analyses and coordinates referenced here are for the mm9 mouse reference genome URLs. UCSC Genome Browser Track Hub containing normalised replicate ATAC-seq and normalised NG Capture-C data without windowing: http://sara.molbiol.ox.ac/public/hugheslab/Primitive_alpha_globin/hub.txt .

Files in database submission

Fastq files

Genome browser session
(e.g. UCSC)

No longer applicable for final submission documents.

Methodology

Replicates

Typically, a minimum of three biological replicates; number of replicates have been indicated for each experiment

Sequencing depth	Libraries were submitted to between 9 and 11 cycles of PCR (NEBNext kit). Insert size was verified to be 250bp by Agilent Tapestation. Libraries were quantified using the KAPA library quantification kit (KAPA Biosystems) and then sequenced on the NextSeq platform. All experiments contained >40m reads obtained using High-output 75 cycle kits (Illumina), paired end sequencing.
Antibodies	CTCF Millipore 07-729<br>H3K27ac Abcam ab4729<br>H3K4me1 Merck 07-436<br>H3K4me3 AbCam ab8580<br>H2A119ub Cell Signaling technology #8240 D27C4<br>H3K27me3 Cell Signaling technology #9733<br>GATA1 AbCam ab11852<br>All antibodies used have previously been published in ChIP-seq studies.
Peak calling parameters	All generated data were not used for genome wide analyses, but only to show local enrichment at the alpha-globin locus. ChIP-seq data were therefore verified by direct visual inspection at the appropriate locus on the UCSC genome browser.
Data quality	ChIP enrichment was verified by qPCR prior to sequencing (>5 fold). Quality of sequencing was verified by FastQC.
Software	Sequencing quality was assessed by FASTQC 0.11.4. ChIP-seq and ATAC-seq data were aligned to the mm9 (for mouse) and hg19 (for human) data using a customised in house pipeline (script: https://github.com/Hughes-Genome-Group/NGseqBasic/releases). Output BAM files were sorted and indexed using Samtools 0.1.19, then normalised using Deeptools 2.2.2 to RPKM (--normalizeUsingRPKM), then converted to a bigwig using USCS tools 1.0. Data were visualised in the USCS genome browser. Published datasets were downloaded from the NCBI Gene Expression Omnibus (GEO) database. FASTQ files were then processed as described.

Flow Cytometry

Plots

Confirm that:

- [x] The axis labels state the marker and fluorochrome used (e.g. CD4-FITC).
- [x] The axis scales are clearly visible. Include numbers along axes only for bottom left plot of group (a 'group' is an analysis of identical markers).
- [x] All plots are contour plots with outliers or pseudocolor plots.
- [x] A numerical value for number of cells or percentage (with statistics) is provided.

Methodology

Sample preparation	For experiments using primary primitive erythroblasts, cells were isolated from yolk sacs at E10.5. Cells were collected by successive PBS washes followed by spinning at 200xg for 5 mins.<br>For experiments using definitive erythroid cells from fetal liver culture, fetal livers were dissected from mouse embryos and dissociated by syringe in order to obtain single cell suspension. For spleen culture, cells were isolated from adult spleen and dissociated by syringe to obtain a single cell suspension.
Instrument	Cell sorting was performed on a BD FACs ARIA Fusion machine. Analysis was performed on a ThermoFisher Attune NxT machine.
Software	Initial data collection was performed on the proprietary software from BD FACSAria Fusion and ThermoFisher Attune NxT machines. Analysis was performed using BD FloJo software
Cell population abundance	The cell populations used in this study were highly abundant, as shown in Supplementary Figure 1.<br>All sorted samples showed high purity following examination of cytospins post sort.
Gating strategy	For all the samples, the FSC/SSC gate has been drawn in order to encompass events with a high FSC and a low SSC (large size, low granularity). For HUDEP-2 samples, the starting FSC/SSC gate at day 0 has been defined to take into account the change in size and granularity observed during erythroid differentiation. This initial gate was not changed when analysing later time points. For all the samples, doublets have been removed from the analysis using the FSC-Height vs FSC-Area gating strategy. Only live single cells have been considered for the analysis by selecting the Hoechst 33258 negative population only.<br><br>For all the samples used in the study, gating has been done using an unstained sample, as well as “single stain (SS)” samples (where only one antibody or Hoechst is used) and “fluorescence minus one (FMO)” samples (where all antibodies and Hoechst is used, except one) when more than one antibody was used. In order to remove any fluorescence spill-over between channels, compensation has been performed. Gating boundaries were defined using the unstained, SS and FMO controls and remained unchanged when analysing the samples. Gating strategies are shown in Supplementary Figure 8 and Supplementary Figures 14-18.

☒ Tick this box to confirm that a figure exemplifying the gating strategy is provided in the Supplementary Information.

Magnetic resonance imaging

Experimental design

Design type	*Indicate task or resting state; event-related or block design.*
Design specifications	*Specify the number of blocks, trials or experimental units per session and/or subject, and specify the length of each trial or block (if trials are blocked) and interval between trials.*
Behavioral performance measures	*State number and/or type of variables recorded (e.g. correct button press, response time) and what statistics were used to establish that the subjects were performing the task as expected (e.g. mean, range, and/or standard deviation across subjects).*

Acquisition

Imaging type(s)	*Specify: functional, structural, diffusion, perfusion.*
Field strength	*Specify in Tesla*
Sequence & imaging parameters	*Specify the pulse sequence type (gradient echo, spin echo, etc.), imaging type (EPI, spiral, etc.), field of view, matrix size, slice thickness, orientation and TE/TR/flip angle.*
Area of acquisition	*State whether a whole brain scan was used OR define the area of acquisition, describing how the region was determined.*

Diffusion MRI ☐ Used ☐ Not used

Preprocessing

Preprocessing software	*Provide detail on software version and revision number and on specific parameters (model/functions, brain extraction, segmentation, smoothing kernel size, etc.).*
Normalization	*If data were normalized/standardized, describe the approach(es): specify linear or non-linear and define image types used for transformation OR indicate that data were not normalized and explain rationale for lack of normalization.*
Normalization template	*Describe the template used for normalization/transformation, specifying subject space or group standardized space (e.g. original Talairach, MNI305, ICBM152) OR indicate that the data were not normalized.*
Noise and artifact removal	*Describe your procedure(s) for artifact and structured noise removal, specifying motion parameters, tissue signals and physiological signals (heart rate, respiration).*
Volume censoring	*Define your software and/or method and criteria for volume censoring, and state the extent of such censoring.*

Statistical modeling & inference

Model type and settings	*Specify type (mass univariate, multivariate, RSA, predictive, etc.) and describe essential details of the model at the first and second levels (e.g. fixed, random or mixed effects; drift or auto-correlation).*
Effect(s) tested	*Define precise effect in terms of the task or stimulus conditions instead of psychological concepts and indicate whether ANOVA or factorial designs were used.*

Specify type of analysis: ☐ Whole brain ☐ ROI-based ☐ Both

Statistic type for inference (See Eklund et al. 2016)	*Specify voxel-wise or cluster-wise and report all relevant parameters for cluster-wise methods.*
Correction	*Describe the type of correction and how it is obtained for multiple comparisons (e.g. FWE, FDR, permutation or Monte Carlo).*

Models & analysis

n/a	Involved in the study
☐	☐ Functional and/or effective connectivity
☐	☐ Graph analysis
☐	☐ Multivariate modeling or predictive analysis

Functional and/or effective connectivity	*Report the measures of dependence used and the model details (e.g. Pearson correlation, partial correlation, mutual information).*
Graph analysis	*Report the dependent variable and connectivity measure, specifying weighted graph or binarized graph, subject- or group-level, and the global and/or node summaries used (e.g. clustering coefficient, efficiency, etc.).*
Multivariate modeling and predictive analysis	*Specify independent variables, features extraction and dimension reduction, model, training and evaluation metrics.*
